# Characteristics of adverse events and clinical risks of intravenous immunoglobulin: a pharmacovigilance study based on FDA Adverse Event Reporting System (FAERS)

**DOI:** 10.3389/fmed.2025.1724196

**Published:** 2026-01-07

**Authors:** Zhen Lu, Xiaonan Lu, Yao Gao, Guangbin Shang, Yingjian Zeng

**Affiliations:** 1 The Affiliated Hospital of Jiangxi University of Chinese Medicine, Nanchang, China; 2 Research Center for Differentiation and Development of Basic theory of Traditional Chinese Medicine, Nanchang, China; 3 School of Chinese Medicine, Jiangxi University of Chinese Medicine, Nanchang, China

**Keywords:** intravenous immunoglobulin, drug adverse events, FAERS database, pharmacovigilance, drug safety signal detection, disproportionality analysis

## Abstract

**Background:**

Intravenous immunoglobulin (IVIg) is widely used to treat primary immunodeficiency, chronic inflammatory demyelinating polyneuropathy, immune thrombocytopenia, and other disorders. Although effective in maintaining IgG trough levels and reducing infections, its safety profile requires further characterization.

**Methods:**

A large-scale pharmacovigilance study was conducted using the U.S. FDA Adverse Event Reporting System (FAERS) from Q1 2004 to Q4 2024. Four disproportionality methods—reporting odds ratio (ROR), proportional reporting ratio (PRR), Bayesian confidence propagation neural network (BCPNN), and multi-item gamma Poisson shrinker (MGPS)—were applied to detect adverse event signals. Weibull modeling was used to assess temporal risk patterns.

**Results:**

A total of 76,138 IVIg-associated reports were identified. Common events included infusion-site reactions (swelling, erythema, pain), infections (upper respiratory tract infection, bronchitis, pneumonia, influenza, urinary tract infection), and systemic reactions (pyrexia, chills, hypersensitivity, headache, asthenia, nausea, vomiting). Several novel potential safety signals emerged, including blood pressure–related events (hypertension and hypotension), weight changes (loss and gain), and falls.

**Conclusion:**

Real-world FAERS data confirm the established tolerability of IVIg while highlighting rare but clinically important safety signals, particularly hemolytic anemia and aseptic meningitis. These findings warrant further clinical investigation to optimize monitoring and promote safer therapeutic use.

## Background

Intravenous immunoglobulin (IVIg) is a core treatment for primary immunodeficiency (PID), chronic inflammatory demyelinating polyneuropathy (CIDP), immune thrombocytopenia (ITP), and many other diseases. Since receiving market approval, IVIg has been indicated for conditions such as PID and chronic ITP and has demonstrated strong effectiveness in real-world studies (1, 2). Retrospective multicentre cohorts further confirm that IVIg can maintain ideal IgG trough levels and significantly reduce infection rates in both PID and secondary immunodeficiency populations (3). Randomized controlled trials and open-label studies consistently demonstrate that IVIg is typically well-tolerated; common adverse events (AEs), such as headache, fever, chills, and mild skin reactions, typically occur at rates < 20% (4, 5).

However, high-dose or rapid infusion of IVIg can induce rare but potentially fatal hemolytic anemia, especially in patients with blood types A, B, or AB (6, 7). Large case series of transplant recipients and children with Kawasaki disease suggest that non-O blood type, cumulative dose, and high titers of anti-A/B isoagglutinins in the product are major risk factors (6). Analyses of large spontaneous reporting databases have reinforced this understanding: global AE data show a higher reporting rate of IVIg-related hemolysis than those of similar products, implicating high-titer anti-A/B antibodies as a key mechanism (8).

The FDA Adverse Event Reporting System (FAERS) contains over 28 million spontaneous reports submitted by healthcare professionals, patients, and the pharmaceutical industry (9, 10). FAERS can rapidly identify potential safety signals using disproportionality methods such as the reporting odds ratio (ROR) and proportional reporting ratio (PRR) (11). Previous FAERS-based pharmacovigilance studies have identified novel or rare toxicities of biologics, including unexpected respiratory and endocrine events with anti-IL-5 monoclonal antibodies (e.g., asthmatic crisis, adrenal insufficiency) and newly detected safety signals such as alopecia and sepsis associated with ocrelizumab in multiple sclerosis (12, 13). But a systematic FAERS analysis specifically for IVIg is unavailable.

Accordingly, this study aimed to utilize FAERS data from 2004 to 2024 to comprehensively describe the spectrum, signal strength, and temporal trends of IVIg-related AEs, with special focus on serious events such as hemolytic anemia, to provide evidence for clinical risk assessment and individualized therapy.

## Materials and methods

### Data source and processing

FDA Adverse Event Reporting System data from Q1 of 2004 to Q4 of 2024 were collected. Only reports where IVIg was listed as the primary suspected drug were included. Data extraction and preprocessing were performed using R software (version 4.3.2). The initial dataset comprised 15,942,054 reports. After removing 2,204,454 duplicate records per FDA guidelines, key fields such as PRIMARYID, CASEID, and FDA_DATE were extracted. For reports sharing the same CASEID, only the entry with the latest FDA_DATE was retained; if both CASEID and FDA_DATE were identical, the record with the highest PRIMARYID was retained. AEs were coded using Medical Dictionary for Regulatory Activities (MedDRA; version 27.1) preferred terms (PTs) and organized into system organ classes (SOCs).

### Statistical analysis

To identify potential associations between IVIg and AEs, disproportionality analysis was conducted. This key pharmacovigilance tool uses a 2 × 2 contingency table to compare observed frequencies between exposed and non-exposed populations (Supplementary Table 1). Four disproportionality methods were applied to detect drug–AE signals: ROR, PRR, Bayesian confidence propagation neural network (BCPNN), and multi-item gamma Poisson shrinker (MGPS). An advantage of the ROR is its capacity to adjust for bias, particularly in situations where event counts are low (14). PRR offers higher specificity than that of ROR (15). BCPNN is particularly effective at integrating multi-source data and performing cross-validation (16). MGPS can detect signals from rare events (17). The formulas and threshold criteria for these four algorithms are presented in Supplementary Table 2. Statistical analyses were performed using R software. Higher values indicate stronger signal strength, suggesting a higher consistency between the drug and the occurrence of AEs. Subgroup analyses were conducted by repeating the disproportionality analyses within strata defined by sex (male vs. female), age (<18, 18–65, 65–85, >85 years), body weight (<50, 50–100, >100 kg), and reporter type (healthcare professionals vs. non-professionals). For time-to-onset analyses, we fitted a Weibull distribution to the onset-time data using maximum likelihood estimation, obtaining the shape (β) and scale (α) parameters with 95% confidence intervals. The AE was deemed a potential signal only if it surpassed the positive thresholds in all four disproportionality methods (ROR, PRR, BCPNN, and MGPS).

## Results

### Basic characteristics of IVIg-related AEs

**FIGURE 1:**
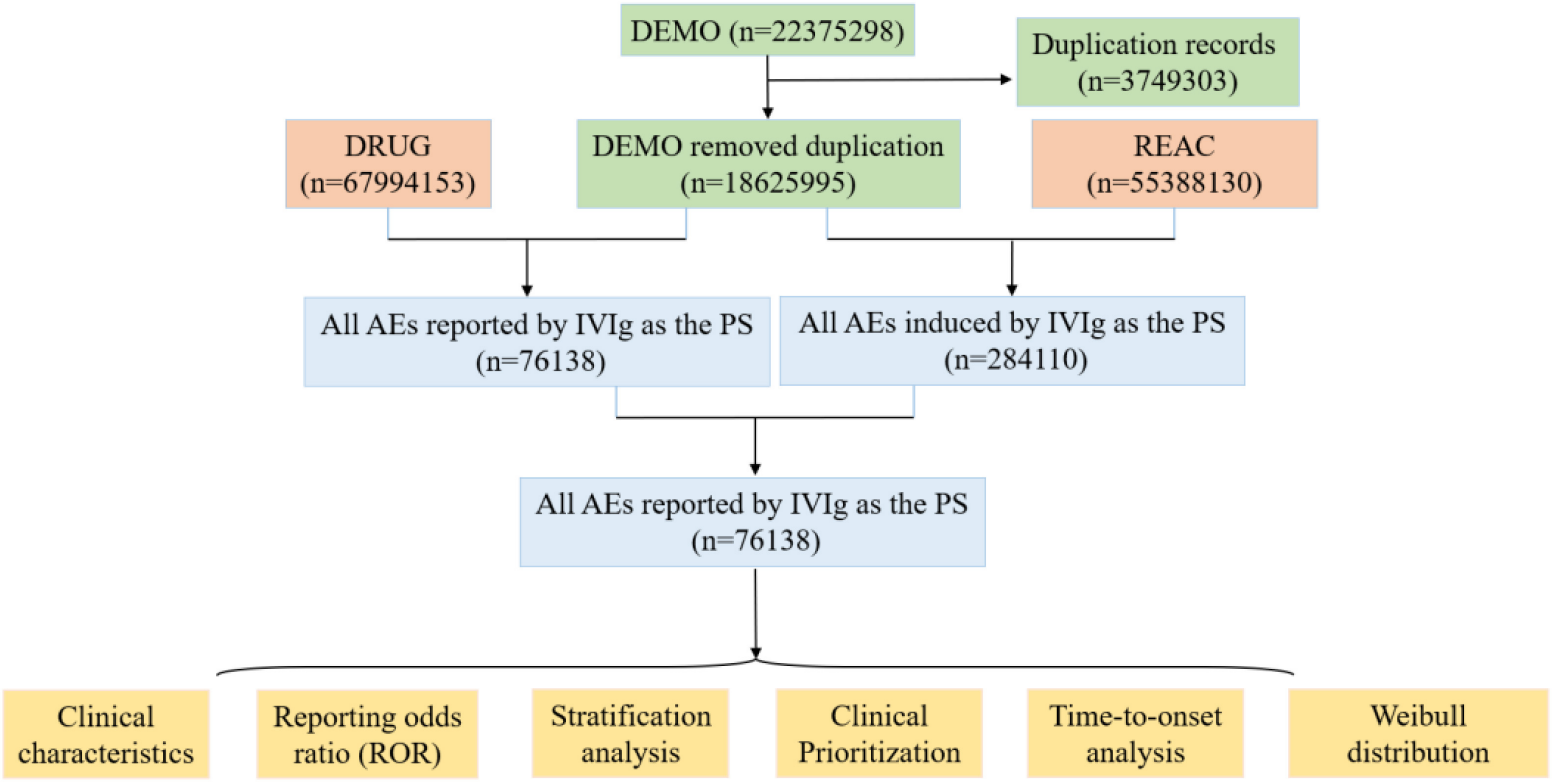
A flowchart illustrates the process of adverse event analysis for intravenous immunoglobulin (IVIg) using the FDA Adverse Event Reporting System database. PS, primary suspect drug.

**FIGURE 2:**
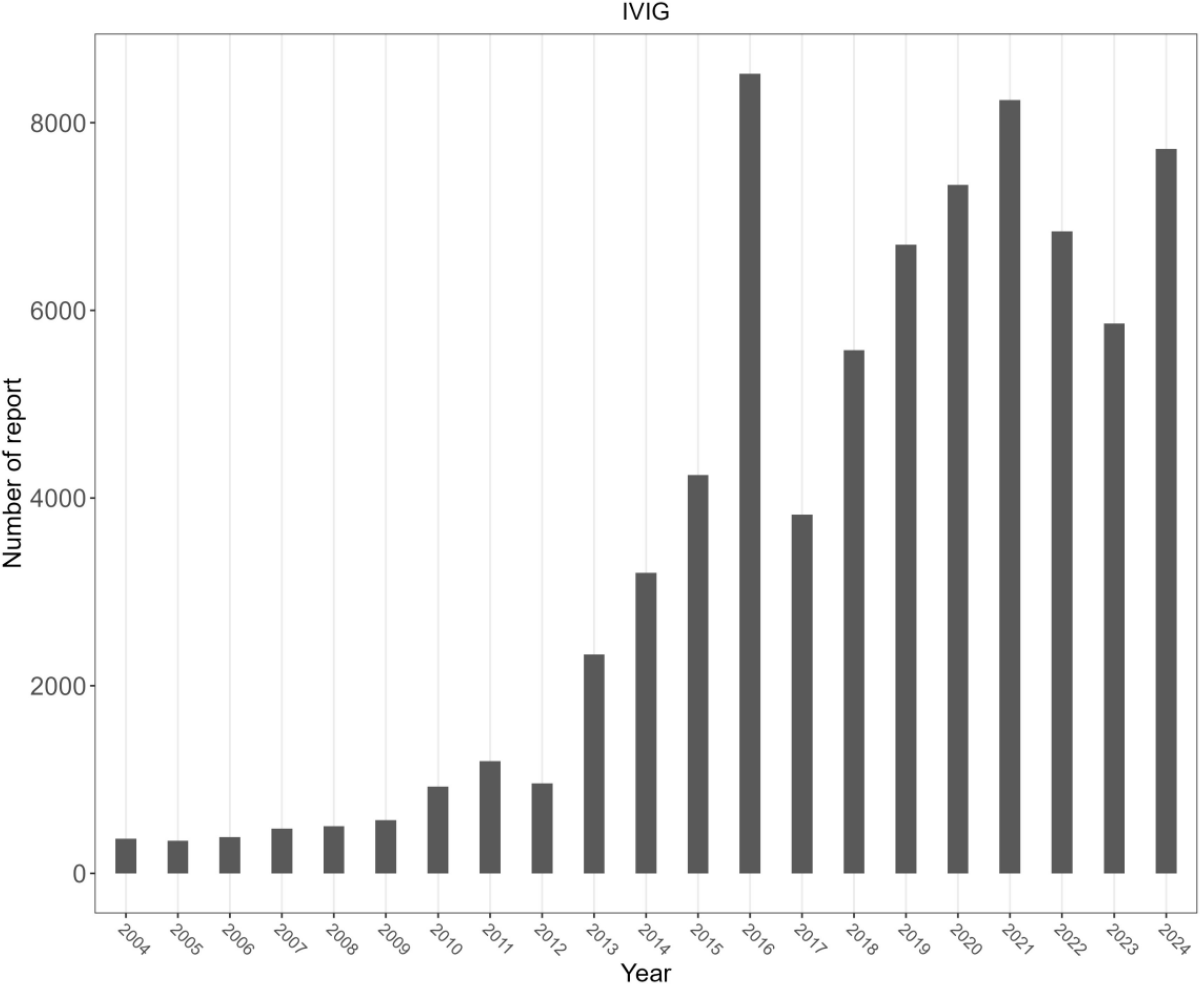
Number of reports per year.

Figure 1 illustrates the identification of IVIg-related AEs from the FAERS database. A total of 76,138 IVIg-related AE reports were identified in FAERS. Overall, women accounted for approximately 58% of reports and adults aged 18–65 years formed the largest age group. The top three countries submitting AE reports were the United States, China, and Japan (Table 1). The annual number of reports attained its peak in 2015, thereafter showing a decrease (Figure 2).

**TABLE 1:** Basic information table.

ID	Count	Percentage
Overall	76,138	–
**Sex**		
F	44,185	58.00%
M	24,912	32.70%
Missing	7,041	9.20%
**Weight**		
<50 kg	4,579	6.00%
50–100 kg	3,924	5.20%
>100 kg	21,882	28.70%
Missing	45,753	60.10%
**Age**		
<18	6,153	8.10%
>85	509	0.70%
18–65	24,761	32.50%
65–85	10,966	14.40%
Missing	33,749	44.30%
**Role**		
CN	22,592	29.70%
HP	14,264	18.70%
LW	32	0.01%
MD	12,279	16.10%
OT	13,868	18.20%
PH	10,234	13.40%

CN, consumer; HP, other health professional; LW, lawyer; MD, physician; OT, other; PH, pharmacist.

### Signal detection at the SOC level

**FIGURE 3:**
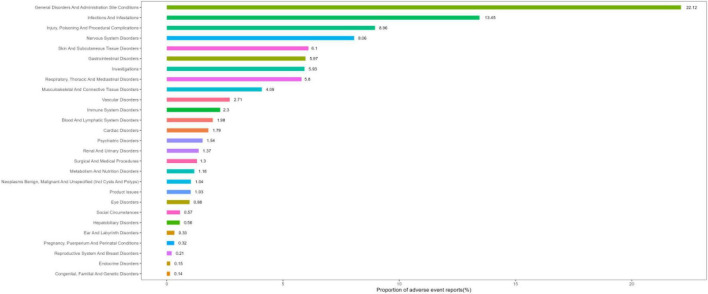
System organ class (SOC) ranking based on percentage of reports.

Figure 3 depicts the proportions of the 27 SOC categories associated with IVIg. A summary of the key results is presented in Table 2, while detailed signal strengths for each SOC category are provided in Supplementary Table 3. The most frequently reported category was “*General disorders and administration site conditions*,” whereas “*Infections and infestations*” had the strongest signal. Several other SOCs also revealed significant signal detection results, including “*Skin and subcutaneous tissue disorders*,” “*Respiratory, thoracic and mediastinal disorders*,” “*Vascular disorders*,” and “*Immune system disorders*.” Categories where the lower limit of the 95% confidence interval for ROR was <1 including “*Injury*,” “*Poisoning and procedural complications*,” “*Nervous system disorders*,” “*Gastrointestinal disorders*,” “*Investigations*,” and “*Musculoskeletal and connective tissue disorders*,” did not reach signal-positive.

**TABLE 2:** Signal strength of intravenous immunoglobulin (IVIg) adverse events (AEs) across SOC in the FDA Adverse Event Reporting System (FAERS) database.

SOC	Number of reports	ROR (95% Cl)	PRR(X^2^)	EBGM (EBGM05)	IC (IC025)
General disorders and administration site conditions	62,849	1.35 (1.33–1.36)	1.27 (4314.92)	1.27 (1.26)	0.34 (0.33)
Infections and infestations	38,226	2.84 (2.81–2.87)	2.59 (38930.02)	2.57 (2.54)	1.36 (1.35)
Injury, poisoning and procedural complications	25,465	0.85 (0.84–0.86)	0.86 (608.59)	0.86 (0.85)	−0.21 (−0.23)
Nervous system disorders	22,891	0.95 (0.93–0.96)	0.95 (64.66)	0.95 (0.94)	−0.07 (−0.09)
Skin and subcutaneous tissue disorders	17,342	1.14 (1.12–1.16)	1.13 (283.24)	1.13 (1.11)	0.18 (0.16)
Gastrointestinal disorders	16,957	0.68 (0.67–0.69)	0.7 (2338.7)	0.7 (0.69)	−0.51 (−0.53)
Investigations	16,856	0.96 (0.95–0.98)	0.97 (21.35)	0.97 (0.95)	-0.05 (-0.07)
Respiratory, thoracic and mediastinal disorders	16,478	1.25 (1.23–1.27)	1.23 (763.58)	1.23 (1.21)	0.3 (0.28)
Musculoskeletal and connective tissue disorders	11,612	0.78 (0.76–0.79)	0.79 (711.67)	0.79 (0.77)	−0.35 (−0.37)
Vascular disorders	7,695	1.28 (1.25–1.31)	1.27 (454.53)	1.27 (1.24)	0.35 (0.31)
Immune system disorders	6,523	2.11 (2.06–2.16)	2.08 (3682.99)	2.07 (2.02)	1.05 (1.02)
Blood and lymphatic system disorders	5,625	1.17 (1.14–1.21)	1.17 (142.22)	1.17 (1.14)	0.23 (0.19)

ROR, reporting odds ratio; PRR, proportional reporting ratio; EBGM, empirical Bayesian geometric mean; EBGM05, the lower limit of the 95% CI of EBGM; IC, information component; IC025, the lower limit of the 95% CI of the IC; CI, confidence interval; SOC, system organ class.

### Signal detection at the PT level

All IVIg-related AEs were ranked according to their frequency of occurrence. The key findings are summarized in Table 3, while the full list of preferred terms is provided in Supplementary Table 4. The top 50 PTs were mainly concentrated in three areas: infusion site events (e.g., infusion site swelling, infusion site erythema, infusion site extravasation, infusion site pain), infection events (e.g., upper respiratory tract infection, bronchitis, pneumonia, influenza, urinary tract infection), and systemic events (e.g., pyrexia, chills, hypersensitivity, headache, asthenia, nausea, vomiting).

**TABLE 3:** Top 50 frequency of adverse events at the preferred term (PT) level.

PT	Number of reports	ROR (95% Cl)	PRR (X2)	EBGM (EBGM05)	IC (IC025)
Headache	7,622	2.71 (2.65–2.77)	2.66 (7885.04)	2.64 (2.58)	1.4 (1.37)
Pyrexia	4,800	3.07 (2.98–3.16)	3.03 (6468.77)	3 (2.91)	1.58 (1.54)
Sinusitis	4,268	9.62 (9.33–9.92)	9.49 (30951.74)	9.09 (8.82)	3.18 (3.14)
No adverse event	3,331	4.27 (4.13–4.42)	4.23 (8076.23)	4.17 (4.02)	2.06 (2.01)
Pneumonia	3,278	2.13 (2.05–2.2)	2.11 (1913.88)	2.1 (2.03)	1.07 (1.02)
Chills	3,081	5.84 (5.63–6.05)	5.78 (11862.88)	5.65 (5.45)	2.5 (2.44)
Urticaria	2,816	3.85 (3.71–4)	3.82 (5769.2)	3.77 (3.63)	1.91 (1.86)
Infusion related reaction	2,717	9.94 (9.56–10.33)	9.85 (20588.52)	9.43 (9.07)	3.24 (3.18)
Covid-19	2,259	2.82 (2.71–2.94)	2.81 (2601.46)	2.78 (2.67)	1.48 (1.41)
Infection	2,121	3.34 (3.2–3.49)	3.33 (3398.67)	3.29 (3.15)	1.72 (1.65)
Infusion site pain	1,805	40.91 (38.88–43.05)	40.66 (57735.7)	33.79 (32.11)	5.08 (4.98)
Infusion site erythema	1,703	71.09 (67.24–75.15)	70.67 (85739.46)	52.06 (49.25)	5.7 (5.58)
Infusion site swelling	1,612	100.78 (94.88–107.03)	100.21 (104401.78)	66.41 (62.53)	6.05 (5.91)
Bronchitis	1,558	4.55 (4.33–4.79)	4.53 (4197.6)	4.45 (4.23)	2.15 (2.08)
Migraine	1,317	3.14 (2.97–3.31)	3.13 (1877.89)	3.09 (2.93)	1.63 (1.55)
Illness	1,154	3.16 (2.98–3.35)	3.15 (1667.78)	3.11 (2.94)	1.64 (1.55)
Upper respiratory tract infection	1,100	5.39 (5.08–5.73)	5.37 (3813.88)	5.26 (4.95)	2.39 (2.3)

ROR, reporting odds ratio; PRR, proportional reporting ratio; EBGM, empirical Bayesian geometric mean; EBGM05, the lower limit of the 95% CI of EBGM; IC, information component; IC025, the lower limit of the 95% CI of the IC; CI, confidence interval; PT, preferred term.

### Subgroup analysis

In the subgroup analysis, several differences emerged. Sex: Pyrexia and headache were commonly reported in both men and women; however, men exhibited a higher incidence of chills and urticaria, whereas women were predisposed to sinusitis and pneumonia. Age: Hemolytic anemia and hemorrhage were more frequently observed in individuals <18 years of age. Weight: Patients weighing >50 kg had higher incidence of urinary tract infection and upper respiratory tract infection, whereas those <50 kg were more susceptible to ear infections. Reporter Type: Healthcare professionals tended to report more serious systemic conditions, such as aseptic meningitis, serum sickness, and chronic inflammatory demyelinating polyradiculoneuropathy. In contrast, reports from non-professionals (patients and caregivers) primarily involved infectious complications and functional impairments. These differences reflect the distinct perspectives of medical professionals and patients or families regarding disease perception and clinical priorities (Supplementary Tables 5–15).

### Time-to-onset and Weibull distribution analysis

**FIGURE 4:**
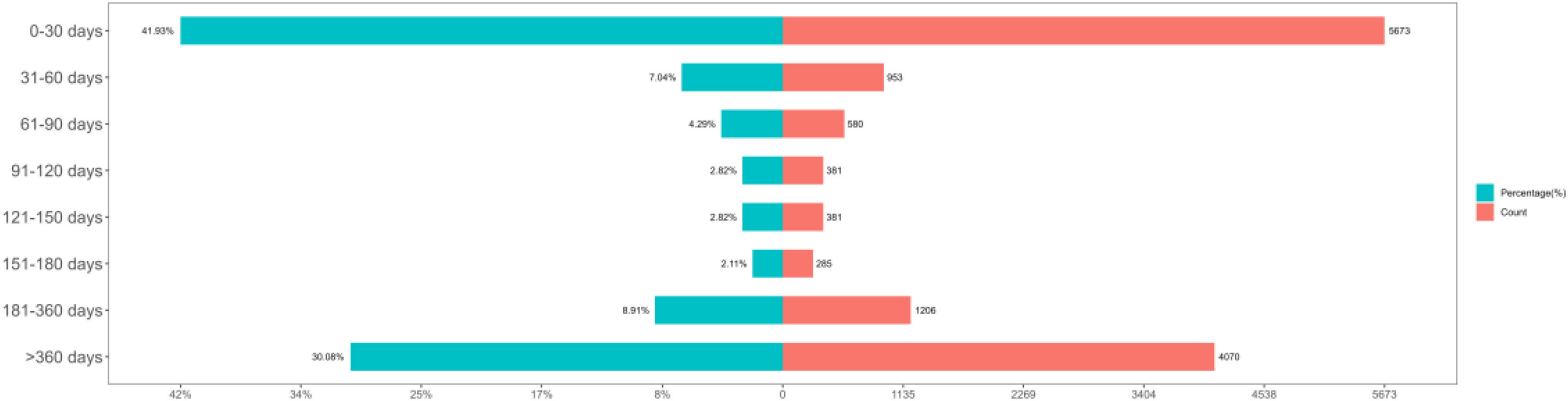
Time-to-onset of adverse events (AEs).

Intravenous immunoglobulin-related AEs most frequently occurred within the first month of treatment. The time-to-onset distribution is shown in Figure 4. The median time to onset was 66 days (interquartile range: 7–523 days), and the Weibull analysis showed an early-failure type (shape parameter β 0.47 [95% CI 0.46–0.47]; scale parameter α 189.41 [95% CI 182.16–196.66]).

### Sensitivity analysis

Intravenous immunoglobulinis frequently used in combination with several other drugs, such as Hizentra, Diphenhydramine, Acetaminophen, Gammagard liquid and Human immunoglobulin G. After excluding reports involving combinations with these six drugs, we included 18,996 reports, which encompassed 4,480 adverse event reports. The adverse reactions that still met the positive criteria (Supplementary Table 16).

## Discussion

In this large pharmacovigilance study based on 76,138 FAERS reports, we systematically characterized the safety profile of IVIg. At the SOC level, “general disorders and administration site conditions” was the most frequently reported category, whereas “infections and infestations” showed the strongest association signal. Infusion-site reactions and systemic infusion-related symptoms (e.g., headache, pyrexia, chills) dominated the PT-level spectrum, and this pattern was broadly consistent across sex, age, weight, and reporter subgroups as well as in the sensitivity analysis excluding common co-medications. This pattern is consistent with IVIg’s clinical applications and known risks (18, 19).

Among the AEs detected in our FAERS analysis, hemolytic anemia emerged as a particularly prominent signal, with a high reporting odds ratio (ROR) and consistent detection across multiple subgroups (age, weight). This finding was unexpected, as it reinforces the known risk of hemolysis in high-dose IVIg therapy, but also highlights a particularly strong signal in certain patient populations. Hemolytic anemia is one of the most serious hematologic complications of IVIg treatment, primarily caused by anti-A and anti-B isoagglutinins present in IVIg products (20). The risk of IVIg-induced hemolysis is strongly associated with blood type: non-O blood types, particularly AB, have a significantly increased risk. High-dose IVIg therapy further increases the risk, particularly in patients with underlying inflammatory conditions (21). Patients with inflammatory diseases, such as Kawasaki disease, have elevated hemolysis rates, possibly owing to enhanced macrophage activation in inflammatory states (22).

Our study also found significant signals for vascular disorders and renal/urinary disorders, suggesting that IVIg-associated kidney injury warrants attention. The mechanisms of IVIg-induced acute kidney injury (AKI) are complex and include osmotic nephrosis, intravascular thrombosis, and immune-mediated injury (23). Osmotic nephrosis is primarily caused by stabilizers in IVIg formulations, such as sucrose or mannitol, which accumulate in renal tubular cells and lead to vacuolization and renal dysfunction (24). Infusion speed and total dose are critical contributors to AKI risk.

For patients with pre-existing renal insufficiency, comprehensive pre-treatment evaluation is advised, including assessment of baseline renal function and hydration status, and the selection of IVIg products free of sucrose or mannitol. During treatment, close monitoring of serum creatinine levels, blood urea nitrogen, and urine analysis is essential, with dose or infusion speed adjustments—or even treatment suspension—if necessary (23).

Intravenous immunoglobulin-induced aseptic meningitis is a rare but important neurologic AE, occurring in approximately 0.42%–0.60% of cases (25). Affected patients typically develop headache, fever, nuchal rigidity, and nausea or vomiting within 24–48 h after administration of IVIg infusion (26). The exact mechanism remains unclear, but may involve type III or type IV hypersensitivity reactions, direct toxicity, or immune complex deposition (27). Cerebrospinal fluid analysis typically reveals lymphocytic pleocytosis, mild protein elevation, normal glucose levels, and negative bacterial cultures (28). Clinically, IVIg-associated aseptic meningitis must be distinguished from infectious meningitis. IVIg-induced meningitis is self-limiting and resolves within 5–7 days after discontinuation of IVIg therapy without the need for specific treatment, although supportive care may be required (29). For patients who must continue IVIg therapy, premedication or switching to an alternative IVIg formulation can be considered.

Skin and subcutaneous tissue disorders also showed significant signals in this study. Common manifestations include rash, pruritus, urticaria, and angioedema. These reactions are typically mild to moderate in severity and may involve histamine release, complement activation, or allergic mechanisms (19).

Thromboembolic events are a well-recognized major concern in IVIg therapy, and the FDA has issued a black box warning to clinicians regarding this risk (30). Thrombosis related to IVIg is believed to result from IgG aggregates that activate platelets, increase blood viscosity, and promote a hypercoagulable state (31). Risk factors include advanced age, history of thrombosis, prolonged immobility, cardiac disease, and malignancy. To prevent thromboembolic complications, clinicians should evaluate patients’ thrombotic risk factors prior to treatment, avoid rapid infusion rates, ensure adequate hydration, and consider prophylactic anticoagulation in high-risk patients (32).

This study utilized FAERS data and applied four disproportionality methods (ROR, PRR, BCPNN, and MGPS) to enhance the sensitivity and specificity of signal detection (33, 34). FAERS, as one of the largest spontaneous reporting systems globally, plays a crucial role in post-marketing safety surveillance. However, spontaneous reports are subject to inherent limitations, including underreporting, selective reporting bias, incomplete information, and duplication (35). Therefore, our findings indicate potential safety signals but do not establish definitive causal relationships. Additionally, differences in reporting practices and healthcare systems across countries may influence the generalizability of our results (36).

This study had several limitations. First, FAERS is a voluntary reporting system that depends on healthcare professionals, patients, and pharmaceutical manufacturers, which may introduce selection bias, including under-reporting, duplicate entries, and incomplete data. Although duplicate records were removed in accordance with FDA guidelines, some missing or inconsistent information may persist. Second, FAERS lacks critical details of IVIg therapy, such as infusion rate, blood type matching, and anti-A/B isoagglutinin titers, which are important confounding variables closely linked to severe adverse reactions such as hemolytic anemia. Third, the database does not capture information on IVIg treatment cycles, dosing intervals, or prior exposure, which are essential factors that may influence the occurrence of adverse events. Additionally, notable regional bias exists, as the majority of reports originate from the United States; this may limit the applicability of our findings to populations with different ethnic, genetic, and disease backgrounds. Variations in clinical practice patterns, reporting habits, and regulatory requirements across regions may also influence the completeness and representativeness of AE reporting. These inherent limitations highlight the need for large-scale prospective cohort studies and randomized controlled trials to validate the safety signals identified in this study and to provide stronger evidence for the individualized and safe use of IVIg.

## Conclusion

Using FAERS data from Q1 of 2004 to Q4 of 2024, we performed large-scale, multi-index disproportionality analyses to systematically map the overall distribution, signal strength, and temporal evolution of IVIg-related AEs. The results indicate that the common adverse reactions of IVIg are consistent with those reported in previous clinical trials and package inserts (predominantly headache, fever, chills, and skin reactions). Importantly, our analysis provided further evidence that hemolytic anemia and aseptic meningitis are rare but clinically significant adverse reactions to IVIg. In addition, previously recognized risks such as thromboembolic events and acute kidney injury remain important clinical considerations during IVIg therapy. These findings enhance our understanding of the potential safety risks associated with IVIg and provide evidence-based guidance for clinicians in pre-treatment risk assessment, infusion protocol selection, and risk management.

## Data Availability

The datasets presented in this study can be found in online repositories. The names of the repository/repositories and accession number(s) can be found in the article/Supplementary material.
