# Supplementary material for: Characteristics of adverse events and clinical risks of intravenous immunoglobulin: a pharmacovigilance study based on FDA Adverse Event Reporting System (FAERS)
Source: Front Med (Lausanne). 2026 Jan 7;12:1724196. doi: 10.3389/fmed.2025.1724196 (PMC12819674; doi:10.3389/fmed.2025.1724196)
Supplement: Supplementary file 1 [file Table_1.DOCX]

**Supplementary Table 1** Two-by-two contingency table for disproportionality analyses.

|  | Target AEs | Other AEs | Total |
| --- | --- | --- | --- |
| Target drugs | a | b | a+b |
| Other drugs | c | d | c+d |
| Total | a+c | b+d | a+b+c+d |

Abbreviation: AEs, adverse events; a, number of reports containing both the target drug and target adverse drug reaction; b, number of reports containing other adverse drug reaction of the target drug; c, number of reports containing the target adverse drug reaction of other drugs; d, number of reports containing other drugs and other adverse drug reactions.

**Supplementary Table 2** Four major algorithms used for signal detection.

| Algorithms | Calculation Formula | Threshold |
| --- | --- | --- |
| ROR | $\begin{matrix} ROR=\frac{(a/c)}{(b/d)}=\frac{ad}{bc} \\ SE(InROR)=\sqrt{\left(\frac{1}{a}+\frac{1}{b}+\frac{1}{c}+\frac{1}{d}\right)} \\ 95\%CI=eIn(ROR)\pm1.96\sqrt{\left(\frac{1}{a}+\frac{1}{b}+\frac{1}{c}+\frac{1}{d}\right)} \end{matrix}$ | a≥3 with a lower 95% CI > 1 |
| PRR | $\begin{matrix} PRR=\frac{\text{a}/\left(a+b\right)}{c/(c+d)} \\ SE(InPRR)=\sqrt{\frac{1}{a}-\frac{1}{a+b}+\frac{1}{c}-\frac{1}{c+d}} \\ 95\%CI=eIn(PRR)\pm1.96\sqrt{(\frac{1}{a}-\frac{1}{a+b}+\frac{1}{c}-\frac{1}{c+d})} \end{matrix}$ | a≥3 with a lower 95% CI > 1 |
| BCPNN | α1=β1=1; α=β=2; γ11=1 | (-): E(IC)≤0  (+):0< E(IC) |
| MGPS |  | EBGM05>2, a>0 |

Abbreviation: a number of reports containing both the target drug and target adverse drug reaction; b, number of reports containing other adverse drug reaction of the target drug; c, number of reports containing the target adverse drug reaction of other drugs; d, number of reports containing other drugs and other adverse drug reactions. 95%CI, 95% confidence interval; N, the number of reports; χ2, chi-squared; IC, information component; IC025, the lower limit of 95% CI of the IC; E(IC), the IC expectations; EBGM, empirical Bayesian geometric mean; EBGM05, the lower limit of 95% CI of EBGM.

**Supplementary Table 3**

Signal strength of IVIg AEs across SOC in the FAERS database.

| SOC | Number of reports | ROR(95%Cl) | PRR(X^2^) | EBGM(EBGM05) | IC(IC025) |
| --- | --- | --- | --- | --- | --- |
| General disorders and administration site conditions | 62849 | 1.35(1.33-1.36) | 1.27(4314.92) | 1.27(1.26) | 0.34(0.33) |
| Infections and infestations | 38226 | 2.84(2.81-2.87) | 2.59(38930.02) | 2.57(2.54) | 1.36(1.35) |
| Injury, poisoning and procedural complications | 25465 | 0.85(0.84-0.86) | 0.86(608.59) | 0.86(0.85) | -0.21(-0.23) |
| Nervous system disorders | 22891 | 0.95(0.93-0.96) | 0.95(64.66) | 0.95(0.94) | -0.07(-0.09) |
| Skin and subcutaneous tissue disorders | 17342 | 1.14(1.12-1.16) | 1.13(283.24) | 1.13(1.11) | 0.18(0.16) |
| Gastrointestinal disorders | 16957 | 0.68(0.67-0.69) | 0.7(2338.7) | 0.7(0.69) | -0.51(-0.53) |
| Investigations | 16856 | 0.96(0.95-0.98) | 0.97(21.35) | 0.97(0.95) | -0.05(-0.07) |
| Respiratory, thoracic and mediastinal disorders | 16478 | 1.25(1.23-1.27) | 1.23(763.58) | 1.23(1.21) | 0.3(0.28) |
| Musculoskeletal and connective tissue disorders | 11612 | 0.78(0.76-0.79) | 0.79(711.67) | 0.79(0.77) | -0.35(-0.37) |
| Vascular disorders | 7695 | 1.28(1.25-1.31) | 1.27(454.53) | 1.27(1.24) | 0.35(0.31) |
| Immune system disorders | 6523 | 2.11(2.06-2.16) | 2.08(3682.99) | 2.07(2.02) | 1.05(1.02) |
| Blood and lymphatic system disorders | 5625 | 1.17(1.14-1.21) | 1.17(142.22) | 1.17(1.14) | 0.23(0.19) |
| Cardiac disorders | 5081 | 0.67(0.65-0.69) | 0.68(794.71) | 0.68(0.66) | -0.56(-0.6) |
| Psychiatric disorders | 4389 | 0.26(0.25-0.27) | 0.27(8963.82) | 0.27(0.27) | -1.87(-1.91) |
| Renal and urinary disorders | 3896 | 0.71(0.69-0.74) | 0.72(437.47) | 0.72(0.7) | -0.48(-0.52) |
| Surgical and medical procedures | 3704 | 0.97(0.94-1) | 0.97(4.35) | 0.97(0.94) | -0.05(-0.1) |
| Metabolism and nutrition disorders | 3353 | 0.54(0.52-0.56) | 0.54(1315.25) | 0.54(0.53) | -0.88(-0.93) |
| Neoplasms benign, malignant and unspecified (incl cysts and polyps) | 2942 | 0.39(0.37-0.4) | 0.39(2830.21) | 0.39(0.38) | -1.34(-1.4) |
| Product issues | 2940 | 0.64(0.61-0.66) | 0.64(598.62) | 0.64(0.62) | -0.64(-0.69) |
| Eye disorders | 2778 | 0.48(0.47-0.5) | 0.49(1502.9) | 0.49(0.47) | -1.03(-1.08) |
| Social circumstances | 1621 | 1.23(1.17-1.29) | 1.23(67.72) | 1.23(1.17) | 0.29(0.22) |
| Hepatobiliary disorders | 1603 | 0.61(0.58-0.64) | 0.61(397.83) | 0.61(0.58) | -0.71(-0.78) |
| Ear and labyrinth disorders | 947 | 0.77(0.72-0.82) | 0.77(65.61) | 0.77(0.72) | -0.38(-0.47) |

ROR, reporting odds ratio; PRR, proportional reporting ratio; EBGM, empirical Bayesian geometric mean; EBGM05, the lower limit of the 95% CI of EBGM; IC, information component; IC025, the lower limit of the 95% CI of the IC; CI, confidence interval; SOC, System Organ Class.

**Supplementary Table 4**

Top 50 frequency of adverse events at the PT level.

| PT | Number of reports | ROR(95%Cl) | PRR(X2) | EBGM(EBGM05) | IC(IC025) |
| --- | --- | --- | --- | --- | --- |
| Headache | 7622 | 2.71(2.65-2.77) | 2.66(7885.04) | 2.64(2.58) | 1.4(1.37) |
| Pyrexia | 4800 | 3.07(2.98-3.16) | 3.03(6468.77) | 3(2.91) | 1.58(1.54) |
| Sinusitis | 4268 | 9.62(9.33-9.92) | 9.49(30951.74) | 9.09(8.82) | 3.18(3.14) |
| No adverse event | 3331 | 4.27(4.13-4.42) | 4.23(8076.23) | 4.17(4.02) | 2.06(2.01) |
| Pneumonia | 3278 | 2.13(2.05-2.2) | 2.11(1913.88) | 2.1(2.03) | 1.07(1.02) |
| Chills | 3081 | 5.84(5.63-6.05) | 5.78(11862.88) | 5.65(5.45) | 2.5(2.44) |
| Urticaria | 2816 | 3.85(3.71-4) | 3.82(5769.2) | 3.77(3.63) | 1.91(1.86) |
| Infusion related reaction | 2717 | 9.94(9.56-10.33) | 9.85(20588.52) | 9.43(9.07) | 3.24(3.18) |
| Covid-19 | 2259 | 2.82(2.71-2.94) | 2.81(2601.46) | 2.78(2.67) | 1.48(1.41) |
| Infection | 2121 | 3.34(3.2-3.49) | 3.33(3398.67) | 3.29(3.15) | 1.72(1.65) |
| Infusion site pain | 1805 | 40.91(38.88-43.05) | 40.66(57735.7) | 33.79(32.11) | 5.08(4.98) |
| Infusion site erythema | 1703 | 71.09(67.24-75.15) | 70.67(85739.46) | 52.06(49.25) | 5.7(5.58) |
| Infusion site swelling | 1612 | 100.78(94.88-107.03) | 100.21(104401.78) | 66.41(62.53) | 6.05(5.91) |
| Bronchitis | 1558 | 4.55(4.33-4.79) | 4.53(4197.6) | 4.45(4.23) | 2.15(2.08) |
| Migraine | 1317 | 3.14(2.97-3.31) | 3.13(1877.89) | 3.09(2.93) | 1.63(1.55) |
| Illness | 1154 | 3.16(2.98-3.35) | 3.15(1667.78) | 3.11(2.94) | 1.64(1.55) |
| Upper respiratory tract infection | 1100 | 5.39(5.08-5.73) | 5.37(3813.88) | 5.26(4.95) | 2.39(2.3) |
| Meningitis aseptic | 1084 | 74.57(69.52-79.99) | 74.29(56675.35) | 53.99(50.34) | 5.75(5.59) |
| Tachycardia | 1022 | 2.51(2.36-2.67) | 2.51(916.92) | 2.49(2.34) | 1.32(1.22) |
| Infusion site extravasation | 978 | 41.98(39.17-44.99) | 41.84(32069.69) | 34.59(32.28) | 5.11(4.96) |
| Infusion site pruritus | 934 | 134.33(123.57-146.02) | 133.89(72883.73) | 79.62(73.24) | 6.32(6.09) |
| Influenza like illness | 913 | 2.36(2.21-2.52) | 2.36(706.7) | 2.34(2.19) | 1.23(1.13) |
| Anaphylactic reaction | 882 | 3.68(3.44-3.93) | 3.67(1684.09) | 3.62(3.39) | 1.86(1.75) |
| Ear infection | 849 | 7.52(7.02-8.05) | 7.5(4605.34) | 7.26(6.78) | 2.86(2.75) |
| Drug ineffective for unapproved indication | 843 | 3.47(3.24-3.72) | 3.46(1452.05) | 3.42(3.19) | 1.77(1.67) |
| Insurance issue | 793 | 17.77(16.52-19.11) | 17.72(11467.72) | 16.32(15.18) | 4.03(3.89) |
| Oxygen saturation decreased | 704 | 2.88(2.67-3.1) | 2.87(848.09) | 2.85(2.64) | 1.51(1.4) |
| Respiratory tract infection | 625 | 5.64(5.21-6.11) | 5.63(2313.29) | 5.5(5.08) | 2.46(2.33) |
| Therapy interrupted | 581 | 2.27(2.1-2.47) | 2.27(409.29) | 2.26(2.08) | 1.17(1.05) |
| Viral infection | 568 | 3.92(3.61-4.26) | 3.91(1207.77) | 3.85(3.55) | 1.95(1.82) |
| Infusion site bruising | 556 | 110.59(99.65-122.73) | 110.38(38407.06) | 70.71(63.71) | 6.14(5.83) |
| Haemolytic anaemia | 554 | 14.12(12.95-15.39) | 14.09(6281.8) | 13.2(12.11) | 3.72(3.56) |
| Infusion site haemorrhage | 533 | 53.36(48.48-58.73) | 53.26(21444.07) | 42(38.16) | 5.39(5.15) |
| Incorrect drug administration rate | 513 | 33.7(30.68-37.01) | 33.64(13844.9) | 28.81(26.23) | 4.85(4.64) |
| Body temperature increased | 470 | 4.97(4.53-5.45) | 4.96(1450.4) | 4.86(4.44) | 2.28(2.14) |
| Haemolysis | 459 | 13.65(12.42-15.01) | 13.63(5019.35) | 12.8(11.64) | 3.68(3.5) |
| Staphylococcal infection | 405 | 2.62(2.37-2.89) | 2.62(399.17) | 2.59(2.35) | 1.38(1.23) |
| Hypoxia | 390 | 2.46(2.22-2.71) | 2.45(332.1) | 2.44(2.2) | 1.28(1.13) |
| Poor venous access | 387 | 9.11(8.22-10.08) | 9.09(2663.79) | 8.73(7.89) | 3.13(2.95) |
| Recalled product administered | 351 | 12.73(11.42-14.18) | 12.71(3554.46) | 11.99(10.76) | 3.58(3.38) |
| Cystitis | 349 | 2.34(2.11-2.6) | 2.34(265.3) | 2.33(2.09) | 1.22(1.06) |
| Seasonal allergy | 347 | 5.94(5.34-6.61) | 5.93(1381.22) | 5.79(5.2) | 2.53(2.36) |
| Gastroenteritis viral | 345 | 4.36(3.92-4.85) | 4.36(872.61) | 4.28(3.85) | 2.1(1.93) |
| Sinus disorder | 334 | 3.75(3.37-4.18) | 3.75(660.07) | 3.69(3.32) | 1.89(1.71) |
| Serum sickness | 331 | 28.65(25.53-32.16) | 28.62(7689.08) | 25.07(22.34) | 4.65(4.38) |
| Infusion site reaction | 328 | 36.39(32.34-40.95) | 36.35(9497.01) | 30.77(27.35) | 4.94(4.65) |
| Infusion site mass | 328 | 146.08(126.57-168.59) | 145.91(26939.14) | 83.7(72.52) | 6.39(5.87) |
| Blood immunoglobulin g decreased | 328 | 39.96(35.48-45) | 39.91(10319.65) | 33.27(29.54) | 5.06(4.75) |
| Feeling cold | 324 | 2.52(2.26-2.82) | 2.52(293.86) | 2.5(2.24) | 1.32(1.16) |
| Multiple allergies | 314 | 9.52(8.5-10.66) | 9.51(2279.64) | 9.11(8.14) | 3.19(2.98) |

ROR, reporting odds ratio; PRR, proportional reporting ratio; EBGM, empirical Bayesian geometric mean; EBGM05, the lower limit of the 95% CI of EBGM; IC, information component; IC025, the lower limit of the 95% CI of the IC; CI, confidence interval; PT, preferred term.

**Supplementary Table 5**

Top 50 most frequent adverse events for IVIg at the PT level in males from FAERS data.

| PT | Number of reports | ROR(95%Cl) | PRR(X2) | EBGM(EBGM05) | IC(IC025) |
| --- | --- | --- | --- | --- | --- |
| Pyrexia* | 1793 | 3.26(3.11-3.41) | 3.21(2706.22) | 3.18(3.03) | 1.67(1.6) |
| Headache* | 1634 | 2.54(2.42-2.67) | 2.51(1484.77) | 2.5(2.38) | 1.32(1.25) |
| Chills* | 1240 | 7.4(6.99-7.84) | 7.31(6546.7) | 7.1(6.71) | 2.83(2.74) |
| Urticaria* | 1016 | 5.96(5.59-6.34) | 5.9(4031.35) | 5.77(5.42) | 2.53(2.43) |
| Pruritus* | 1003 | 2.19(2.06-2.33) | 2.18(634.15) | 2.16(2.03) | 1.11(1.02) |
| Infusion related reaction* | 895 | 12.94(12.09-13.84) | 12.81(9208.19) | 12.15(11.35) | 3.6(3.49) |
| No adverse event* | 878 | 5.09(4.76-5.45) | 5.05(2794.09) | 4.96(4.64) | 2.31(2.2) |
| Sinusitis* | 866 | 10.22(9.55-10.95) | 10.13(6814.15) | 9.72(9.08) | 3.28(3.17) |
| Infection* | 599 | 3.24(2.99-3.51) | 3.23(907.95) | 3.19(2.94) | 1.67(1.55) |
| Blood pressure increased* | 465 | 2.22(2.03-2.44) | 2.22(307.62) | 2.2(2.01) | 1.14(1) |
| Tachycardia* | 442 | 3.37(3.07-3.7) | 3.36(722.04) | 3.32(3.02) | 1.73(1.59) |
| Haemorrhage* | 432 | 2.52(2.29-2.77) | 2.51(389.95) | 2.5(2.27) | 1.32(1.17) |
| Infusion site pain* | 416 | 44.04(39.62-48.94) | 43.83(14465.98) | 36.58(32.91) | 5.19(4.92) |
| Drug ineffective for unapproved indication* | 367 | 5.26(4.74-5.83) | 5.24(1230.36) | 5.14(4.63) | 2.36(2.19) |
| Infusion site erythema* | 354 | 64.04(56.87-72.12) | 63.79(16876.39) | 49.43(43.89) | 5.63(5.27) |
| Infusion site swelling* | 324 | 84.19(74.04-95.74) | 83.88(19091.98) | 60.63(53.32) | 5.92(5.5) |
| Oxygen saturation decreased* | 318 | 3.81(3.41-4.26) | 3.8(646.26) | 3.75(3.36) | 1.91(1.73) |
| Anaphylactic reaction* | 303 | 4.61(4.12-5.17) | 4.6(836.68) | 4.53(4.04) | 2.18(1.99) |
| Infusion site extravasation* | 278 | 42.46(37.33-48.3) | 42.33(9374.58) | 35.53(31.24) | 5.15(4.8) |
| Bronchitis* | 245 | 3.37(2.97-3.83) | 3.37(401.79) | 3.33(2.94) | 1.74(1.54) |
| Upper respiratory tract infection* | 244 | 4.91(4.32-5.57) | 4.9(740.28) | 4.81(4.24) | 2.27(2.06) |
| Ear infection* | 234 | 9.04(7.93-10.3) | 9.01(1600.72) | 8.69(7.63) | 3.12(2.88) |
| Meningitis aseptic* | 229 | 44.06(38.22-50.8) | 43.95(7981.97) | 36.66(31.8) | 5.2(4.78) |
| Illness* | 228 | 2.86(2.51-3.26) | 2.86(272.02) | 2.83(2.49) | 1.5(1.3) |
| Haemolytic anaemia* | 217 | 14.19(12.37-16.29) | 14.16(2490.69) | 13.35(11.63) | 3.74(3.46) |
| Hypoxia* | 202 | 3.16(2.75-3.63) | 3.16(293.37) | 3.12(2.72) | 1.64(1.42) |
| Haemolysis* | 197 | 15.42(13.34-17.82) | 15.38(2473.09) | 14.42(12.48) | 3.85(3.54) |
| Insurance issue* | 190 | 14.49(12.51-16.79) | 14.46(2231.79) | 13.62(11.75) | 3.77(3.46) |
| Body temperature increased* | 186 | 5.93(5.13-6.86) | 5.92(740.54) | 5.79(5) | 2.53(2.28) |
| Serum sickness* | 184 | 59.3(50.36-69.81) | 59.17(8253.44) | 46.62(39.6) | 5.54(4.99) |
| Therapy interrupted* | 183 | 2.41(2.08-2.79) | 2.41(148.8) | 2.39(2.07) | 1.26(1.03) |
| Migraine* | 180 | 3.34(2.89-3.88) | 3.34(290.66) | 3.3(2.85) | 1.72(1.49) |
| Injection site swelling* | 164 | 2.6(2.23-3.04) | 2.6(159.55) | 2.58(2.21) | 1.37(1.13) |
| Infusion site pruritus* | 164 | 143.7(117.92-175.11) | 143.43(13917.76) | 86.46(70.95) | 6.43(5.56) |
| Respiratory tract infection* | 163 | 5.11(4.37-5.97) | 5.1(525.27) | 5.01(4.29) | 2.32(2.06) |
| Viral infection* | 155 | 3.77(3.21-4.41) | 3.76(308.9) | 3.71(3.17) | 1.89(1.63) |
| Haemarthrosis* | 152 | 6.78(5.77-7.97) | 6.77(724.58) | 6.59(5.61) | 2.72(2.43) |
| Tachypnoea* | 140 | 5.44(4.6-6.43) | 5.43(493.68) | 5.32(4.5) | 2.41(2.12) |
| Cytomegalovirus infection* | 140 | 4.57(3.87-5.41) | 4.57(381.91) | 4.49(3.8) | 2.17(1.89) |
| Incorrect drug administration rate* | 138 | 28.73(24.05-34.32) | 28.69(3253.88) | 25.43(21.29) | 4.67(4.17) |
| Infusion site bruising* | 129 | 140.38(112.45-175.25) | 140.17(10793.28) | 85.27(68.3) | 6.41(5.39) |
| Infusion site haemorrhage* | 128 | 54.83(45.15-66.58) | 54.75(5384.27) | 43.85(36.11) | 5.45(4.76) |
| Chronic inflammatory demyelinating polyradiculoneuropathy* | 122 | 60.01(49.09-73.35) | 59.93(5529.18) | 47.09(38.52) | 5.56(4.81) |
| Feeling cold* | 110 | 3.27(2.7-3.94) | 3.26(170.08) | 3.23(2.67) | 1.69(1.39) |
| Acute graft versus host disease in skin* | 109 | 20.98(17.23-25.55) | 20.96(1887.77) | 19.19(15.76) | 4.26(3.75) |
| Acute graft versus host disease* | 104 | 13.89(11.39-16.93) | 13.87(1167.03) | 13.09(10.74) | 3.71(3.26) |
| Lip swelling* | 100 | 2.48(2.03-3.02) | 2.48(87.1) | 2.46(2.02) | 1.3(0.99) |
| Factor viii inhibition* | 100 | 29.34(23.81-36.17) | 29.31(2406.78) | 25.92(21.03) | 4.7(4.07) |
| Poor venous access* | 97 | 10.14(8.27-12.43) | 10.13(762) | 9.72(7.92) | 3.28(2.86) |
| Transplant rejection* | 94 | 6.77(5.51-8.31) | 6.76(447.5) | 6.59(5.36) | 2.72(2.34) |

Abbreviation: Asterisks (*) indicate relevant signal in algorithm; ROR, reporting odds ratio; PRR, proportional reporting ratio; EBGM, empirical Bayesian geometric mean; EBGM05, the lower limit of the 95% CI of EBGM; IC, information component; IC025, the lower limit of the 95% CI of the IC; CI, confidence interval; PT, preferred term; AEs, adverse events.

**Supplementary Table 6**

Top 50 most frequent adverse events for IVIg at the PT level in females from FAERS data.

| PT | Number of reports | ROR (95%Cl) | PRR(X2) | EBGM(EBGM05) | IC(IC025) |
| --- | --- | --- | --- | --- | --- |
| Headache* | 5610 | 2.66(2.59-2.73) | 2.6(5527.25) | 2.58(2.51) | 1.37(1.33) |
| Sinusitis* | 3317 | 9.19(8.87-9.52) | 9.04(22581.86) | 8.64(8.34) | 3.11(3.06) |
| Pyrexia* | 2704 | 2.9(2.79-3.02) | 2.87(3268.86) | 2.84(2.74) | 1.51(1.45) |
| Pneumonia* | 2118 | 2.38(2.28-2.49) | 2.37(1655.01) | 2.35(2.25) | 1.23(1.17) |
| No adverse event* | 1998 | 5(4.78-5.23) | 4.96(6149) | 4.85(4.63) | 2.28(2.21) |
| Urticaria* | 1683 | 3.15(3-3.31) | 3.13(2408.36) | 3.1(2.95) | 1.63(1.56) |
| Infusion related reaction* | 1632 | 9.54(9.07-10.03) | 9.46(11721.2) | 9.02(8.58) | 3.17(3.09) |
| Chills* | 1625 | 4.75(4.52-4.99) | 4.71(4636.35) | 4.61(4.39) | 2.21(2.13) |
| Covid-19* | 1612 | 3.19(3.03-3.35) | 3.17(2352.69) | 3.13(2.98) | 1.64(1.57) |
| Infection* | 1424 | 3.72(3.53-3.92) | 3.7(2750.35) | 3.64(3.45) | 1.86(1.78) |
| Urinary tract infection* | 1386 | 2.26(2.15-2.39) | 2.25(957.59) | 2.24(2.12) | 1.16(1.08) |
| Infusion site pain* | 1309 | 38.27(36.04-40.64) | 38(38677.25) | 31.34(29.51) | 4.97(4.85) |
| Infusion site erythema* | 1287 | 69.05(64.72-73.67) | 68.56(61375.22) | 49.39(46.29) | 5.63(5.48) |
| Bronchitis* | 1286 | 4.73(4.47-5) | 4.7(3654.08) | 4.6(4.35) | 2.2(2.12) |
| Infusion site swelling* | 1227 | 103.81(96.71-111.43) | 103.11(77752.55) | 64.98(60.54) | 6.02(5.85) |
| Migraine* | 1089 | 3(2.82-3.19) | 2.99(1418.17) | 2.95(2.78) | 1.56(1.47) |
| Illness* | 908 | 3.45(3.23-3.69) | 3.44(1545.08) | 3.39(3.18) | 1.76(1.66) |
| Upper respiratory tract infection* | 836 | 5.61(5.23-6.01) | 5.58(3050.92) | 5.44(5.08) | 2.44(2.33) |
| Meningitis aseptic* | 748 | 93.5(85.53-102.2) | 93.11(44315.8) | 60.88(55.7) | 5.93(5.69) |
| Infusion site pruritus* | 739 | 126.37(114.93-138.95) | 125.85(52993.11) | 73.28(66.65) | 6.2(5.93) |
| Influenza like illness* | 672 | 2.36(2.19-2.55) | 2.36(517.73) | 2.34(2.17) | 1.22(1.11) |
| Infusion site extravasation* | 647 | 56.03(51.27-61.23) | 55.83(26344.31) | 42.46(38.85) | 5.41(5.19) |
| Ear infection* | 597 | 6.97(6.42-7.57) | 6.95(2926.3) | 6.72(6.19) | 2.75(2.61) |
| Insurance issue* | 587 | 20.49(18.81-22.32) | 20.42(9700.32) | 18.37(16.86) | 4.2(4.03) |
| Anaphylactic reaction* | 493 | 3.28(3-3.58) | 3.27(763.84) | 3.23(2.95) | 1.69(1.55) |
| Respiratory tract infection* | 449 | 6.24(5.68-6.85) | 6.22(1900.76) | 6.04(5.5) | 2.59(2.44) |
| Infusion site bruising* | 413 | 108.52(95.95-122.73) | 108.27(27000.28) | 66.98(59.23) | 6.07(5.68) |
| Viral infection* | 390 | 3.95(3.57-4.37) | 3.94(838.71) | 3.88(3.51) | 1.96(1.8) |
| Infusion site haemorrhage* | 390 | 50.62(45.21-56.67) | 50.51(14650.61) | 39.32(35.12) | 5.3(5) |
| Ill-defined disorder* | 383 | 2.92(2.64-3.24) | 2.92(476.1) | 2.89(2.61) | 1.53(1.37) |
| Drug ineffective for unapproved indication* | 362 | 2.56(2.31-2.85) | 2.56(339.88) | 2.54(2.29) | 1.34(1.19) |
| Oxygen saturation decreased* | 349 | 2.37(2.14-2.64) | 2.37(273.15) | 2.35(2.12) | 1.23(1.07) |
| Incorrect drug administration rate* | 341 | 41.37(36.75-46.56) | 41.29(10823.73) | 33.53(29.79) | 5.07(4.76) |
| Cystitis* | 315 | 2.48(2.21-2.77) | 2.47(272.54) | 2.45(2.19) | 1.29(1.12) |
| Seasonal allergy* | 294 | 6.81(6.06-7.65) | 6.8(1399.5) | 6.58(5.86) | 2.72(2.52) |
| Poor venous access* | 274 | 9.59(8.49-10.83) | 9.58(1995.29) | 9.13(8.08) | 3.19(2.97) |
| Sinus disorder* | 270 | 3.98(3.53-4.5) | 3.98(589.2) | 3.91(3.47) | 1.97(1.78) |
| Multiple allergies* | 270 | 10.09(8.92-11.41) | 10.08(2086.43) | 9.58(8.47) | 3.26(3.03) |
| Staphylococcal infection* | 268 | 2.96(2.62-3.34) | 2.96(341.4) | 2.92(2.59) | 1.55(1.36) |
| Recalled product administered* | 265 | 20.87(18.37-23.71) | 20.84(4468) | 18.71(16.47) | 4.23(3.95) |
| Haemolytic anaemia* | 262 | 13.04(11.5-14.78) | 13.02(2704.04) | 12.18(10.74) | 3.61(3.36) |
| Gastroenteritis viral* | 260 | 4.37(3.86-4.94) | 4.36(657.42) | 4.28(3.78) | 2.1(1.9) |
| Body temperature increased* | 259 | 4.38(3.87-4.96) | 4.38(658.44) | 4.29(3.8) | 2.1(1.9) |
| Kidney infection* | 256 | 3.47(3.07-3.93) | 3.47(441.5) | 3.42(3.02) | 1.77(1.58) |
| Infusion site mass* | 248 | 162.76(136.85-193.58) | 162.54(20530.17) | 84.29(70.88) | 6.4(5.76) |
| Infusion site reaction* | 235 | 39.15(33.98-45.1) | 39.1(7116.24) | 32.08(27.84) | 5(4.62) |
| Blood immunoglobulin g decreased* | 229 | 46.84(40.47-54.2) | 46.78(8076.39) | 37.04(32) | 5.21(4.79) |
| Haemolysis* | 213 | 14.1(12.26-16.21) | 14.08(2394.13) | 13.1(11.39) | 3.71(3.43) |
| Infusion site nodule* | 192 | 147.81(121.91-179.2) | 147.65(15089.74) | 80.13(66.09) | 6.32(5.57) |
| Photophobia* | 191 | 3.34(2.89-3.85) | 3.33(306.42) | 3.29(2.85) | 1.72(1.49) |

Abbreviation: Asterisks (*) indicate relevant signal in algorithm; ROR, reporting odds ratio; PRR, proportional reporting ratio; EBGM, empirical Bayesian geometric mean; EBGM05, the lower limit of the 95% CI of EBGM; IC, information component; IC025, the lower limit of the 95% CI of the IC; CI, confidence interval; PT, preferred term; AEs, adverse events.

**Supplementary Table 7**

Top 50 most frequent adverse events for IVIg at the PT level in patients aged <18 from FAERS data.

| PT | Number of reports | ROR (95%Cl) | PRR(X2) | EBGM(EBGM05) | IC(IC025) |
| --- | --- | --- | --- | --- | --- |
| Headache* | 857 | 3.3(3.08-3.53) | 3.22(1280.58) | 3.14(2.93) | 1.65(1.55) |
| Sinusitis* | 606 | 3.1(2.86-3.37) | 3.05(815.13) | 2.98(2.75) | 1.58(1.45) |
| Pyrexia* | 292 | 2.99(2.66-3.36) | 2.97(370.51) | 2.91(2.58) | 1.54(1.36) |
| Pneumonia* | 275 | 2.49(2.21-2.81) | 2.48(237.3) | 2.44(2.16) | 1.29(1.1) |
| No adverse event* | 274 | 10.72(9.45-12.15) | 10.61(2144.93) | 9.63(8.5) | 3.27(3.04) |
| Urticaria* | 210 | 6.06(5.27-6.97) | 6.02(827.11) | 5.72(4.97) | 2.52(2.28) |
| Infusion related reaction* | 176 | 2.41(2.07-2.8) | 2.4(139.95) | 2.36(2.03) | 1.24(1.01) |
| Chills* | 155 | 65.59(53.42-80.55) | 65.18(5780.02) | 38.87(31.65) | 5.28(4.69) |
| Covid-19* | 148 | 7.15(6.04-8.45) | 7.11(723.04) | 6.68(5.65) | 2.74(2.44) |
| Infection* | 138 | 21.33(17.72-25.67) | 21.22(2168.58) | 17.49(14.53) | 4.13(3.7) |
| Urinary tract infection* | 126 | 2.61(2.19-3.12) | 2.6(121.42) | 2.56(2.14) | 1.36(1.08) |
| Infusion site pain* | 125 | 33(26.91-40.47) | 32.84(2858.35) | 24.58(20.04) | 4.62(4.08) |
| Infusion site erythema* | 125 | 3.62(3.03-4.33) | 3.61(227.5) | 3.51(2.94) | 1.81(1.52) |
| Bronchitis* | 121 | 2.82(2.36-3.39) | 2.82(137.73) | 2.76(2.3) | 1.47(1.18) |
| Infusion site swelling* | 120 | 47.71(38.29-59.45) | 47.48(3625.67) | 31.86(25.57) | 4.99(4.36) |
| Migraine* | 112 | 3.03(2.51-3.66) | 3.02(146.75) | 2.96(2.45) | 1.56(1.26) |
| Illness* | 111 | 3.22(2.67-3.9) | 3.21(164) | 3.14(2.6) | 1.65(1.35) |
| Upper respiratory tract infection* | 103 | 2.61(2.15-3.18) | 2.61(99.5) | 2.56(2.11) | 1.36(1.05) |
| Meningitis aseptic* | 100 | 4.93(4.03-6.03) | 4.92(296.59) | 4.72(3.86) | 2.24(1.89) |
| Infusion site pruritus* | 96 | 19.95(16-24.87) | 19.87(1420.19) | 16.57(13.29) | 4.05(3.52) |
| Influenza like illness* | 93 | 53.06(41.14-68.43) | 52.86(3026.36) | 34.17(26.49) | 5.09(4.31) |
| Infusion site extravasation* | 91 | 3.22(2.61-3.97) | 3.21(134.09) | 3.14(2.54) | 1.65(1.31) |
| Ear infection* | 83 | 29.58(23.11-37.87) | 29.48(1737.95) | 22.67(17.71) | 4.5(3.82) |
| Insurance issue* | 74 | 5.67(4.48-7.17) | 5.65(267.34) | 5.39(4.26) | 2.43(2) |
| Anaphylactic reaction* | 72 | 3.44(2.72-4.35) | 3.43(119.86) | 3.35(2.64) | 1.74(1.35) |
| Respiratory tract infection* | 68 | 3.4(2.66-4.33) | 3.39(110.59) | 3.31(2.59) | 1.72(1.32) |
| Infusion site bruising* | 67 | 16.62(12.82-21.56) | 16.58(833.63) | 14.24(10.98) | 3.83(3.2) |
| Viral infection* | 67 | 3.42(2.68-4.37) | 3.41(110.49) | 3.33(2.61) | 1.74(1.33) |
| Infusion site haemorrhage* | 61 | 7.93(6.1-10.3) | 7.91(339.82) | 7.37(5.68) | 2.88(2.36) |
| Ill-defined disorder* | 59 | 4.78(3.68-6.22) | 4.77(167.59) | 4.59(3.53) | 2.2(1.73) |
| Drug ineffective for unapproved indication* | 58 | 3.52(2.71-4.57) | 3.51(100.49) | 3.42(2.63) | 1.77(1.33) |
| Oxygen saturation decreased* | 57 | 6.19(4.73-8.1) | 6.18(232.26) | 5.86(4.48) | 2.55(2.04) |
| Incorrect drug administration rate* | 54 | 2.97(2.27-3.9) | 2.97(68.3) | 2.91(2.22) | 1.54(1.09) |
| Cystitis* | 50 | 3.33(2.51-4.41) | 3.32(78.38) | 3.24(2.44) | 1.7(1.22) |
| Seasonal allergy* | 48 | 98.05(65.42-146.95) | 97.86(2251.99) | 48.4(32.29) | 5.6(4.11) |
| Poor venous access* | 48 | 3.63(2.72-4.85) | 3.63(87.99) | 3.53(2.64) | 1.82(1.33) |
| Sinus disorder* | 46 | 7.67(5.68-10.37) | 7.66(246.41) | 7.16(5.3) | 2.84(2.22) |
| Multiple allergies* | 44 | 3.12(2.31-4.21) | 3.11(61.16) | 3.05(2.26) | 1.61(1.1) |
| Staphylococcal infection* | 41 | 4.04(2.95-5.52) | 4.03(89.76) | 3.91(2.86) | 1.97(1.41) |
| Recalled product administered* | 41 | 14.21(10.23-19.74) | 14.19(436.62) | 12.46(8.97) | 3.64(2.81) |
| Haemolytic anaemia* | 40 | 6.57(4.76-9.05) | 6.56(176.16) | 6.19(4.49) | 2.63(1.99) |
| Gastroenteritis viral* | 39 | 13.22(9.45-18.49) | 13.2(385.66) | 11.7(8.36) | 3.55(2.72) |
| Body temperature increased* | 38 | 3.01(2.18-4.15) | 3(49.19) | 2.94(2.13) | 1.56(1.02) |
| Kidney infection* | 34 | 3.91(2.77-5.51) | 3.9(70.5) | 3.79(2.69) | 1.92(1.31) |
| Infusion site mass* | 34 | 4.05(2.87-5.7) | 4.04(74.63) | 3.92(2.78) | 1.97(1.35) |
| Infusion site reaction* | 33 | 18.34(12.62-26.63) | 18.31(451.91) | 15.48(10.66) | 3.95(2.9) |
| Blood immunoglobulin g decreased* | 32 | 11.17(7.74-16.12) | 11.16(264.44) | 10.08(6.98) | 3.33(2.45) |
| Haemolysis* | 30 | 3.95(2.74-5.69) | 3.95(63.34) | 3.83(2.66) | 1.94(1.28) |
| Infusion site nodule* | 30 | 2.99(2.08-4.3) | 2.99(38.52) | 2.93(2.04) | 1.55(0.93) |
| Photophobia* | 30 | 4.51(3.13-6.51) | 4.51(78.18) | 4.35(3.01) | 2.12(1.44) |

Abbreviation: Asterisks (*) indicate relevant signal in algorithm; ROR, reporting odds ratio; PRR, proportional reporting ratio; EBGM, empirical Bayesian geometric mean; EBGM05, the lower limit of the 95% CI of EBGM; IC, information component; IC025, the lower limit of the 95% CI of the IC; CI, confidence interval; PT, preferred term; AEs, adverse events.

**Supplementary Table 8**

Top 50 most frequent adverse events for IVIg at the PT level in patients aged 18-65 from FAERS data.

| PT | Number of reports | ROR (95%Cl) | PRR(X2) | EBGM(EBGM05) | IC(IC025) |
| --- | --- | --- | --- | --- | --- |
| Headache* | 3148 | 2.34(2.25-2.42) | 2.3(2312.17) | 2.28(2.2) | 1.19(1.14) |
| Pyrexia* | 2000 | 2.79(2.66-2.91) | 2.75(2216.69) | 2.73(2.61) | 1.45(1.38) |
| Urticaria* | 1355 | 3.97(3.76-4.19) | 3.93(2916.37) | 3.88(3.67) | 1.95(1.87) |
| Sinusitis* | 1334 | 5.97(5.65-6.31) | 5.91(5294.1) | 5.77(5.46) | 2.53(2.44) |
| Chills* | 1257 | 4.89(4.63-5.18) | 4.85(3757.12) | 4.76(4.5) | 2.25(2.16) |
| Infusion related reaction* | 1135 | 8.75(8.24-9.29) | 8.67(7384.47) | 8.35(7.86) | 3.06(2.96) |
| No adverse event* | 1060 | 7.3(6.87-7.77) | 7.24(5506.97) | 7.02(6.6) | 2.81(2.71) |
| Covid-19* | 848 | 2.48(2.32-2.66) | 2.47(735.47) | 2.45(2.29) | 1.29(1.19) |
| Infection* | 747 | 3.25(3.02-3.49) | 3.23(1136.56) | 3.2(2.97) | 1.68(1.57) |
| Infusion site erythema* | 691 | 71.19(65.25-77.68) | 70.76(34900.76) | 52.23(47.86) | 5.71(5.48) |
| Infusion site pain* | 634 | 32.93(30.27-35.83) | 32.75(16720.18) | 28.2(25.92) | 4.82(4.63) |
| Infusion site swelling* | 596 | 95.43(86.52-105.25) | 94.92(37293.72) | 64.23(58.24) | 6.01(5.72) |
| Migraine* | 574 | 2.58(2.37-2.8) | 2.57(543.43) | 2.55(2.35) | 1.35(1.22) |
| Meningitis aseptic* | 525 | 67.03(60.7-74.02) | 66.72(25343.31) | 50(45.28) | 5.64(5.37) |
| Bronchitis* | 514 | 3.52(3.23-3.84) | 3.51(907.34) | 3.47(3.18) | 1.79(1.66) |
| Chest discomfort* | 508 | 2.21(2.03-2.41) | 2.21(331.59) | 2.19(2.01) | 1.13(1) |
| Tachycardia* | 464 | 2.24(2.04-2.45) | 2.23(313.34) | 2.22(2.03) | 1.15(1.01) |
| Illness* | 371 | 3.2(2.89-3.55) | 3.19(550.13) | 3.16(2.85) | 1.66(1.5) |
| Anaphylactic reaction* | 371 | 3.35(3.02-3.71) | 3.34(599.92) | 3.3(2.98) | 1.72(1.56) |
| Infusion site pruritus* | 371 | 116.67(102.59-132.68) | 116.28(26593.37) | 73.3(64.45) | 6.2(5.76) |
| Upper respiratory tract infection* | 362 | 3.81(3.43-4.23) | 3.8(733.45) | 3.75(3.38) | 1.91(1.74) |
| Drug ineffective for unapproved indication* | 360 | 4.03(3.63-4.48) | 4.02(801.47) | 3.96(3.57) | 1.99(1.82) |
| Insurance issue* | 331 | 18.02(16.1-20.17) | 17.97(4858.38) | 16.54(14.78) | 4.05(3.82) |
| Infusion site extravasation* | 278 | 36.14(31.8-41.08) | 36.06(8000.99) | 30.6(26.92) | 4.94(4.6) |
| Oxygen saturation decreased* | 265 | 2.9(2.57-3.27) | 2.9(324.23) | 2.87(2.54) | 1.52(1.33) |
| Ear infection* | 241 | 4.82(4.24-5.47) | 4.81(709.47) | 4.72(4.15) | 2.24(2.03) |
| Haemolytic anaemia* | 221 | 12.86(11.22-14.73) | 12.83(2263.34) | 12.1(10.56) | 3.6(3.33) |
| Respiratory tract infection* | 221 | 5.05(4.42-5.77) | 5.04(697.89) | 4.94(4.32) | 2.3(2.08) |
| Infusion site bruising* | 202 | 84.75(71.85-99.97) | 84.6(11649.1) | 59.36(50.32) | 5.89(5.29) |
| Haemolysis* | 198 | 14.65(12.68-16.93) | 14.62(2338.63) | 13.68(11.84) | 3.77(3.47) |
| Infusion site haemorrhage* | 183 | 43.88(37.38-51.51) | 43.81(6254.55) | 35.97(30.64) | 5.17(4.69) |
| Throat tightness* | 178 | 2.77(2.39-3.22) | 2.77(198.8) | 2.75(2.37) | 1.46(1.23) |
| Hypoxia* | 177 | 2.66(2.29-3.08) | 2.65(179.93) | 2.63(2.27) | 1.4(1.16) |
| Body temperature increased* | 176 | 3.82(3.29-4.43) | 3.82(358.79) | 3.76(3.24) | 1.91(1.67) |
| Serum sickness* | 171 | 35.44(30.11-41.72) | 35.39(4839.33) | 30.12(25.59) | 4.91(4.45) |
| Incorrect drug administration rate* | 168 | 29.09(24.74-34.22) | 29.05(3962.25) | 25.42(21.62) | 4.67(4.24) |
| Viral infection* | 166 | 2.88(2.47-3.36) | 2.88(200.22) | 2.85(2.44) | 1.51(1.27) |
| Cytomegalovirus infection* | 161 | 4.49(3.84-5.26) | 4.49(427) | 4.41(3.77) | 2.14(1.88) |
| Ill-defined disorder* | 157 | 2.59(2.22-3.04) | 2.59(151.58) | 2.57(2.2) | 1.36(1.12) |
| Photophobia* | 153 | 3.79(3.23-4.45) | 3.79(308.15) | 3.74(3.18) | 1.9(1.64) |
| Recalled product administered* | 136 | 20.63(17.28-24.62) | 20.6(2295.08) | 18.74(15.7) | 4.23(3.79) |
| Respiratory distress* | 126 | 2.64(2.22-3.15) | 2.64(126.73) | 2.62(2.2) | 1.39(1.11) |
| Chronic inflammatory demyelinating polyradiculoneuropathy* | 124 | 62.57(51.11-76.6) | 62.51(5687.3) | 47.61(38.89) | 5.57(4.83) |
| Blood immunoglobulin g decreased* | 123 | 37.16(30.65-45.07) | 37.12(3634) | 31.36(25.86) | 4.97(4.37) |
| Poor venous access* | 121 | 5.96(4.97-7.14) | 5.95(483.9) | 5.81(4.84) | 2.54(2.22) |
| Infusion site mass* | 119 | 118.27(94.19-148.5) | 118.14(8617.09) | 74.03(58.96) | 6.21(5.21) |
| Tachypnoea* | 115 | 4.41(3.67-5.31) | 4.41(296.36) | 4.33(3.6) | 2.12(1.8) |
| Kidney infection* | 113 | 3.03(2.52-3.65) | 3.03(151.64) | 3(2.49) | 1.59(1.29) |
| Infusion site reaction* | 113 | 33.57(27.49-40.99) | 33.54(3045.03) | 28.77(23.57) | 4.85(4.24) |
| Photophobia* | 113 | 3.14(2.6-3.78) | 3.13(161.7) | 3.1(2.57) | 1.63(1.33) |

Abbreviation: Asterisks (*) indicate relevant signal in algorithm; ROR, reporting odds ratio; PRR, proportional reporting ratio; EBGM, empirical Bayesian geometric mean; EBGM05, the lower limit of the 95% CI of EBGM; IC, information component; IC025, the lower limit of the 95% CI of the IC; CI, confidence interval; PT, preferred term; AEs, adverse events.

**Supplementary Table 9**

Top 50 most frequent adverse events for IVIg at the PT level in patients aged 65-85 from FAERS data.

| PT | Number of reports | ROR (95%Cl) | PRR(X2) | EBGM(EBGM05) | IC(IC025) |
| --- | --- | --- | --- | --- | --- |
| Headache* | 877 | 2.35(2.2-2.52) | 2.33(664.57) | 2.32(2.17) | 1.21(1.11) |
| Chills* | 844 | 8.6(8.03-9.22) | 8.47(5371.73) | 8.2(7.65) | 3.04(2.92) |
| Pyrexia* | 755 | 2.68(2.49-2.88) | 2.65(771.21) | 2.63(2.45) | 1.4(1.29) |
| Pruritus* | 729 | 2.57(2.38-2.76) | 2.54(679.16) | 2.53(2.35) | 1.34(1.23) |
| Urticaria* | 572 | 6.44(5.92-7) | 6.37(2524.55) | 6.23(5.73) | 2.64(2.5) |
| Infusion related reaction* | 517 | 14.16(12.95-15.48) | 14.02(5889.33) | 13.26(12.12) | 3.73(3.56) |
| Sinusitis* | 471 | 7.69(7.01-8.43) | 7.63(2626.28) | 7.41(6.76) | 2.89(2.73) |
| Covid-19* | 451 | 2.86(2.61-3.14) | 2.84(534.36) | 2.82(2.57) | 1.5(1.35) |
| Blood pressure increased* | 433 | 2.71(2.46-2.98) | 2.7(457.97) | 2.68(2.43) | 1.42(1.27) |
| No adverse event* | 403 | 8.47(7.67-9.36) | 8.41(2538.92) | 8.14(7.37) | 3.03(2.85) |
| Infusion site erythema* | 270 | 77.98(67.89-89.57) | 77.56(15174.27) | 57.93(50.43) | 5.86(5.38) |
| Infection* | 267 | 2.52(2.23-2.84) | 2.51(240.58) | 2.49(2.21) | 1.32(1.13) |
| Bronchitis* | 252 | 3.42(3.02-3.87) | 3.41(422.71) | 3.37(2.98) | 1.75(1.56) |
| Tachycardia* | 238 | 3.79(3.34-4.31) | 3.78(478.89) | 3.73(3.28) | 1.9(1.7) |
| Infusion site swelling* | 236 | 104.99(89.94-122.55) | 104.49(16518.36) | 71.66(61.4) | 6.16(5.57) |
| Hypersensitivity* | 235 | 2.36(2.07-2.68) | 2.35(180.7) | 2.34(2.05) | 1.22(1.03) |
| Infusion site pain* | 223 | 42.19(36.56-48.69) | 42.01(7523.29) | 35.56(30.81) | 5.15(4.74) |
| Oxygen saturation decreased* | 217 | 3.42(2.99-3.91) | 3.41(364.17) | 3.37(2.95) | 1.75(1.54) |
| Illness* | 178 | 2.97(2.56-3.44) | 2.96(228.09) | 2.93(2.53) | 1.55(1.32) |
| Drug ineffective for unapproved indication* | 147 | 4.09(3.47-4.82) | 4.08(336.01) | 4.03(3.42) | 2.01(1.74) |
| Infusion site pruritus* | 134 | 198.82(157.59-250.85) | 198.29(13979.85) | 105.85(83.9) | 6.73(5.59) |
| Upper respiratory tract infection* | 133 | 4.47(3.77-5.31) | 4.47(350.96) | 4.4(3.7) | 2.14(1.85) |
| Anaphylactic reaction* | 132 | 4.21(3.54-5) | 4.2(315.92) | 4.14(3.48) | 2.05(1.76) |
| Influenza like illness* | 122 | 2.91(2.43-3.47) | 2.9(150.17) | 2.88(2.41) | 1.52(1.24) |
| Injection site swelling* | 119 | 4.14(3.46-4.97) | 4.14(277.98) | 4.08(3.4) | 2.03(1.73) |
| Body temperature increased* | 117 | 7.38(6.14-8.87) | 7.37(623.43) | 7.16(5.96) | 2.84(2.5) |
| Hypoxia* | 107 | 2.59(2.14-3.13) | 2.58(102.82) | 2.57(2.12) | 1.36(1.06) |
| Infusion site extravasation* | 103 | 27.8(22.65-34.12) | 27.75(2364.09) | 24.81(20.21) | 4.63(4.04) |
| Feeling cold* | 97 | 3.87(3.17-4.73) | 3.87(202.81) | 3.82(3.12) | 1.93(1.6) |
| Seasonal allergy* | 95 | 10.39(8.46-12.77) | 10.37(769.25) | 9.96(8.11) | 3.32(2.89) |
| Migraine* | 91 | 3.63(2.95-4.47) | 3.63(170.45) | 3.58(2.91) | 1.84(1.5) |
| Multiple allergies* | 90 | 14.83(11.98-18.36) | 14.81(1087.41) | 13.96(11.27) | 3.8(3.3) |
| Infusion site haemorrhage* | 89 | 63.66(50.31-80.56) | 63.55(4272.45) | 49.77(39.33) | 5.64(4.67) |
| Tachypnoea* | 88 | 6.5(5.26-8.04) | 6.49(397.3) | 6.34(5.12) | 2.66(2.27) |
| Respiratory tract infection* | 88 | 3.95(3.2-4.87) | 3.94(189.99) | 3.89(3.15) | 1.96(1.61) |
| Incorrect drug administration rate* | 88 | 46.54(36.99-58.56) | 46.46(3244.62) | 38.68(30.74) | 5.27(4.43) |
| Lower respiratory tract infection* | 88 | 2.69(2.18-3.32) | 2.69(92.42) | 2.67(2.16) | 1.42(1.08) |
| Insurance issue* | 86 | 16.65(13.37-20.72) | 16.62(1175.63) | 15.54(12.48) | 3.96(3.41) |
| Infusion site bruising* | 82 | 157.9(119.05-209.43) | 157.64(7504.02) | 93.1(70.19) | 6.54(5.08) |
| Haemolysis* | 81 | 17.73(14.14-22.24) | 17.71(1183.67) | 16.49(13.15) | 4.04(3.46) |
| Device infusion issue* | 73 | 84.33(64.43-110.36) | 84.2(4367) | 61.54(47.02) | 5.94(4.7) |
| Eczema* | 72 | 3.08(2.44-3.89) | 3.08(99.74) | 3.05(2.42) | 1.61(1.23) |
| Haemolytic anaemia* | 72 | 8.79(6.94-11.12) | 8.78(477.64) | 8.49(6.7) | 3.08(2.6) |
| Respiratory distress* | 71 | 2.69(2.13-3.4) | 2.69(74.58) | 2.67(2.11) | 1.42(1.04) |
| Ear infection* | 68 | 5.8(4.56-7.38) | 5.8(263.13) | 5.68(4.46) | 2.5(2.06) |
| Recalled product administered* | 66 | 24.25(18.81-31.26) | 24.22(1326.34) | 21.96(17.03) | 4.46(3.69) |
| Blood immunoglobulin g decreased* | 60 | 48.43(36.64-64.02) | 48.37(2290.92) | 39.99(30.25) | 5.32(4.2) |
| Infusion site discharge* | 59 | 125.34(91.16-172.35) | 125.19(4669.82) | 80.79(58.75) | 6.34(4.68) |
| Viral infection* | 57 | 2.66(2.05-3.46) | 2.66(58.46) | 2.64(2.03) | 1.4(0.98) |
| Infusion site mass* | 57 | 207.02(144.47-296.67) | 206.79(6081.86) | 108.22(75.52) | 6.76(4.78) |

Abbreviation: Asterisks (*) indicate relevant signal in algorithm; ROR, reporting odds ratio; PRR, proportional reporting ratio; EBGM, empirical Bayesian geometric mean; EBGM05, the lower limit of the 95% CI of EBGM; IC, information component; IC025, the lower limit of the 95% CI of the IC; CI, confidence interval; PT, preferred term; AEs, adverse events.

**Supplementary Table 10**

Top 50 most frequent adverse events for IVIg at the PT level in patients aged >85 from FAERS data.

| PT | Number of reports | ROR (95%Cl) | PRR(X2) | EBGM(EBGM05) | IC(IC025) |
| --- | --- | --- | --- | --- | --- |
| Chills* | 58 | 20.05(15.37-26.15) | 19.6(984.88) | 18.87(14.47) | 4.24(3.47) |
| Pyrexia* | 42 | 4.6(3.39-6.25) | 4.54(115.24) | 4.51(3.32) | 2.17(1.61) |
| Hypertension* | 35 | 4.06(2.91-5.68) | 4.02(79.02) | 3.99(2.86) | 2(1.4) |
| Urticaria* | 34 | 11.6(8.23-16.33) | 11.45(317.07) | 11.21(7.96) | 3.49(2.62) |
| Blood pressure increased* | 27 | 4.08(2.79-5.97) | 4.04(61.48) | 4.02(2.74) | 2.01(1.31) |
| Back pain* | 26 | 3.38(2.29-4.98) | 3.35(42.78) | 3.34(2.26) | 1.74(1.06) |
| Infusion related reaction* | 25 | 24.5(16.36-36.68) | 24.25(530.98) | 23.14(15.46) | 4.53(3.06) |
| No adverse event* | 24 | 8.23(5.48-12.34) | 8.15(148.33) | 8.04(5.36) | 3.01(2.06) |
| Oxygen saturation decreased* | 22 | 6.91(4.53-10.54) | 6.85(108.59) | 6.77(4.44) | 2.76(1.83) |
| Erythema* | 20 | 3.45(2.22-5.37) | 3.43(34.34) | 3.42(2.2) | 1.77(0.98) |
| Covid-19* | 20 | 3.54(2.28-5.5) | 3.52(35.86) | 3.5(2.25) | 1.81(1.01) |
| Tachycardia* | 18 | 6.45(4.05-10.29) | 6.41(81.27) | 6.34(3.98) | 2.67(1.64) |
| Hypersensitivity* | 15 | 4.3(2.58-7.15) | 4.28(37.37) | 4.25(2.55) | 2.09(1.09) |
| Tachypnoea* | 14 | 16.19(9.49-27.62) | 16.1(192.01) | 15.62(9.16) | 3.97(2.22) |
| Infusion site erythema* | 11 | 106.77(55.52-205.34) | 106.29(940.47) | 87.3(45.4) | 6.45(2.5) |
| Anaphylactic reaction* | 10 | 9.96(5.32-18.65) | 9.92(78.64) | 9.74(5.2) | 3.28(1.56) |
| Infusion site pain* | 9 | 101.5(49.42-208.43) | 101.12(737.81) | 83.79(40.8) | 6.39(2.18) |
| Hypervolaemia* | 8 | 9.39(4.66-18.92) | 9.36(58.61) | 9.2(4.56) | 3.2(1.29) |
| Infusion site swelling* | 8 | 176.27(78.4-396.31) | 175.69(1019.03) | 129.11(57.42) | 7.01(1.99) |
| Anaphylactoid reaction* | 8 | 96.95(45.33-207.33) | 96.63(630.95) | 80.69(37.73) | 6.33(1.98) |
| Respiratory rate increased* | 8 | 19.1(9.41-38.76) | 19.04(131.58) | 18.36(9.05) | 4.2(1.66) |
| Influenza like illness* | 7 | 9.61(4.54-20.32) | 9.58(52.77) | 9.41(4.45) | 3.23(1.16) |
| Infusion site discomfort* | 7 | 847.95(248.06-2898.54) | 845.5(2147.12) | 308.09(90.13) | 8.27(1.65) |
| Body temperature increased* | 7 | 10.31(4.87-21.81) | 10.28(57.43) | 10.09(4.77) | 3.33(1.2) |
| Chronic lymphocytic leukaemia* | 7 | 35.33(16.38-76.18) | 35.23(217) | 32.9(15.26) | 5.04(1.66) |
| Flushing* | 6 | 4.98(2.23-11.15) | 4.97(18.86) | 4.93(2.2) | 2.3(0.56) |
| Cyanosis* | 6 | 7.73(3.45-17.33) | 7.71(34.5) | 7.6(3.39) | 2.93(0.86) |
| Sinusitis* | 6 | 5.59(2.5-12.5) | 5.57(22.28) | 5.52(2.47) | 2.47(0.65) |
| Respiratory distress* | 6 | 4.63(2.07-10.36) | 4.62(16.89) | 4.59(2.05) | 2.2(0.5) |
| Haemolytic anaemia* | 6 | 20.9(9.22-47.38) | 20.86(108.74) | 20.03(8.84) | 4.32(1.31) |
| Infusion site extravasation* | 6 | 27.67(12.14-63.07) | 27.61(145.56) | 26.17(11.48) | 4.71(1.38) |
| Lymphoma* | 5 | 9.34(3.85-22.66) | 9.33(36.48) | 9.17(3.78) | 3.2(0.76) |
| Feeling cold* | 5 | 4.94(2.04-11.93) | 4.93(15.51) | 4.89(2.02) | 2.29(0.38) |
| Back disorder* | 5 | 9.96(4.1-24.17) | 9.94(39.41) | 9.76(4.02) | 3.29(0.79) |
| Infusion site pruritus* | 5 | 201.72(71.01-573.04) | 201.31(703.48) | 142.4(50.13) | 7.15(1.17) |
| Localised infection* | 5 | 5.2(2.15-12.57) | 5.2(16.76) | 5.15(2.13) | 2.36(0.42) |
| Coombs direct test positive* | 5 | 484.14(140.07-1673.43) | 483.14(1202.88) | 242.07(70.03) | 7.92(1.09) |
| Chorea* | 4 | 27.26(9.95-74.7) | 27.22(95.64) | 25.82(9.42) | 4.69(0.78) |
| Ear infection* | 4 | 13.63(5.04-36.85) | 13.61(45.46) | 13.26(4.91) | 3.73(0.63) |
| Hip arthroplasty* | 4 | 8.72(3.24-23.45) | 8.71(26.8) | 8.57(3.19) | 3.1(0.46) |
| Infusion site rash* | 4 | 215.08(66.19-698.91) | 214.73(589.12) | 148.97(45.84) | 7.22(0.78) |
| Sputum discoloured* | 4 | 10.81(4.01-29.15) | 10.8(34.78) | 10.58(3.93) | 3.4(0.55) |
| Incorrect drug administration rate* | 4 | 41.18(14.83-114.4) | 41.12(144.3) | 37.97(13.67) | 5.25(0.82) |
| Pulse absent* | 3 | 9.18(2.93-28.8) | 9.17(21.44) | 9.02(2.88) | 3.17(0.12) |
| Panic attack* | 3 | 7.84(2.5-24.56) | 7.83(17.6) | 7.73(2.47) | 2.95(0.07) |
| Poor venous access* | 3 | 21.66(6.81-68.91) | 21.63(56.51) | 20.75(6.52) | 4.37(0.32) |
| Pharyngeal swelling* | 3 | 14.96(4.74-47.23) | 14.94(37.86) | 14.52(4.6) | 3.86(0.25) |
| Prinzmetal angina* | 3 | 241.87(60.45-967.68) | 241.57(479.16) | 161.38(40.34) | 7.33(0.26) |
| Infusion site warmth* | 3 | 1451.23(150.9-13956.89) | 1449.43(1085.58) | 363.11(37.76) | 8.5(0.05) |
| Livedo reticularis* | 3 | 31.55(9.8-101.5) | 31.51(83.2) | 29.64(9.21) | 4.89(0.36) |

Abbreviation: Asterisks (*) indicate relevant signal in algorithm; ROR, reporting odds ratio; PRR, proportional reporting ratio; EBGM, empirical Bayesian geometric mean; EBGM05, the lower limit of the 95% CI of EBGM; IC, information component; IC025, the lower limit of the 95% CI of the IC; CI, confidence interval; PT, preferred term; AEs, adverse events.

**Supplementary Table 11**

Top 50 most frequent adverse events for IVIg at the PT level in patients with body weight <50kg from FAERS data.

| PT | Number of reports | ROR (95%Cl) | PRR(X2) | EBGM(EBGM05) | IC(IC025) |
| --- | --- | --- | --- | --- | --- |
| Pyrexia* | 630 | 2.98(2.75-3.23) | 2.92(777.52) | 2.86(2.64) | 1.51(1.39) |
| Headache* | 485 | 2.99(2.73-3.28) | 2.94(606.33) | 2.88(2.63) | 1.53(1.38) |
| Sinusitis* | 237 | 12.57(10.97-14.42) | 12.44(2180.42) | 10.99(9.59) | 3.46(3.2) |
| Chills* | 219 | 5.84(5.09-6.7) | 5.79(814.56) | 5.49(4.78) | 2.46(2.22) |
| Urticaria* | 200 | 3.86(3.35-4.45) | 3.83(402.29) | 3.71(3.22) | 1.89(1.66) |
| Infusion related reaction* | 199 | 6.68(5.78-7.73) | 6.63(884.46) | 6.23(5.39) | 2.64(2.39) |
| Ear infection* | 134 | 9.41(7.87-11.25) | 9.35(902.43) | 8.53(7.14) | 3.09(2.75) |
| Tachycardia* | 129 | 2.72(2.28-3.24) | 2.71(135.2) | 2.66(2.23) | 1.41(1.13) |
| Infection* | 118 | 3.18(2.64-3.82) | 3.17(169.06) | 3.09(2.57) | 1.63(1.33) |
| No adverse event* | 117 | 2.95(2.45-3.54) | 2.93(144.63) | 2.87(2.39) | 1.52(1.23) |
| Infusion site pain* | 116 | 19.53(15.96-23.9) | 19.42(1654.49) | 16.03(13.1) | 4(3.54) |
| Covid-19* | 115 | 4.43(3.67-5.34) | 4.41(288.52) | 4.24(3.51) | 2.08(1.77) |
| Nasopharyngitis* | 115 | 2.65(2.2-3.19) | 2.64(113.54) | 2.59(2.15) | 1.37(1.08) |
| Infusion site erythema* | 110 | 25.93(20.95-32.11) | 25.8(2017.85) | 20.08(16.22) | 4.33(3.79) |
| Upper respiratory tract infection* | 100 | 5.59(4.57-6.85) | 5.57(352.56) | 5.29(4.32) | 2.4(2.05) |
| Meningitis aseptic* | 92 | 84.62(63.46-112.84) | 84.24(3824.53) | 43.07(32.3) | 5.43(4.52) |
| Influenza* | 91 | 2.78(2.26-3.43) | 2.77(100.14) | 2.72(2.2) | 1.44(1.11) |
| Infusion site swelling* | 91 | 34.66(27.16-44.21) | 34.5(2113.64) | 24.92(19.53) | 4.64(3.96) |
| Haemolytic anaemia* | 77 | 19(14.84-24.33) | 18.93(1072.44) | 15.7(12.26) | 3.97(3.37) |
| Acute graft versus host disease in skin* | 75 | 98.17(70.5-136.7) | 97.8(3363.99) | 46.31(33.26) | 5.53(4.44) |
| Infusion site extravasation* | 72 | 32.22(24.57-42.25) | 32.11(1580.69) | 23.66(18.04) | 4.56(3.79) |
| Factor viii inhibition* | 69 | 24.93(19.07-32.6) | 24.85(1225.71) | 19.51(14.92) | 4.29(3.56) |
| Migraine* | 66 | 3.3(2.58-4.22) | 3.29(101.5) | 3.21(2.51) | 1.68(1.27) |
| Infusion site pruritus* | 66 | 69.5(50.24-96.13) | 69.27(2460.8) | 38.83(28.07) | 5.28(4.2) |
| Bronchitis* | 64 | 3.02(2.35-3.87) | 3.01(83.09) | 2.94(2.29) | 1.56(1.15) |
| Haemolysis* | 64 | 21.58(16.41-28.39) | 21.52(1001.84) | 17.41(13.24) | 4.12(3.4) |
| Cytomegalovirus test positive* | 64 | 36.59(27.31-49.03) | 36.48(1551.22) | 25.92(19.34) | 4.7(3.81) |
| Body temperature increased* | 64 | 6.08(4.72-7.84) | 6.07(253.11) | 5.73(4.45) | 2.52(2.05) |
| Viral infection* | 63 | 3.35(2.6-4.31) | 3.34(99.68) | 3.26(2.53) | 1.7(1.28) |
| Anaphylactic reaction* | 62 | 3.55(2.75-4.58) | 3.54(108.81) | 3.44(2.67) | 1.78(1.36) |
| Respiratory tract infection* | 61 | 4.86(3.75-6.29) | 4.85(176.48) | 4.64(3.59) | 2.21(1.75) |
| Cytomegalovirus infection* | 48 | 6.88(5.12-9.23) | 6.86(222.73) | 6.43(4.79) | 2.68(2.1) |
| Staphylococcal infection* | 45 | 3.47(2.57-4.68) | 3.46(75.88) | 3.37(2.5) | 1.75(1.24) |
| Insurance issue* | 43 | 21.31(15.26-29.77) | 21.27(666.11) | 17.25(12.35) | 4.11(3.17) |
| Tachypnoea* | 43 | 2.85(2.1-3.86) | 2.84(49.83) | 2.79(2.06) | 1.48(0.98) |
| Incorrect drug administration rate* | 41 | 23.42(16.58-33.08) | 23.37(690.51) | 18.59(13.16) | 4.22(3.22) |
| Acute graft versus host disease* | 39 | 35.78(24.62-51.98) | 35.71(930) | 25.53(17.57) | 4.67(3.46) |
| Cytomegalovirus viraemia* | 38 | 24.27(16.93-34.79) | 24.23(660.38) | 19.12(13.34) | 4.26(3.19) |
| Bacterial infection* | 38 | 4.18(3.02-5.79) | 4.18(87.61) | 4.03(2.91) | 2.01(1.43) |
| Chronic graft versus host disease* | 37 | 93.83(58.89-149.51) | 93.66(1624.36) | 45.37(28.48) | 5.5(3.8) |
| Gastroenteritis viral* | 36 | 4.94(3.53-6.92) | 4.93(106.83) | 4.72(3.37) | 2.24(1.61) |
| Product availability issue* | 36 | 7.07(5.03-9.93) | 7.06(173.05) | 6.6(4.7) | 2.72(2.02) |
| Epstein-barr virus infection* | 35 | 13.97(9.77-19.97) | 13.95(362.03) | 12.14(8.49) | 3.6(2.69) |
| Poor venous access* | 33 | 7.99(5.59-11.42) | 7.98(184.37) | 7.39(5.17) | 2.88(2.12) |
| Acute graft versus host disease in intestine* | 33 | 69.39(43.86-109.76) | 69.27(1230.35) | 38.83(24.55) | 5.28(3.59) |
| Thrombotic microangiopathy* | 32 | 7.49(5.22-10.76) | 7.48(165.43) | 6.97(4.85) | 2.8(2.03) |
| Staphylococcal sepsis* | 31 | 10.16(7-14.74) | 10.14(228.64) | 9.18(6.33) | 3.2(2.33) |
| Seasonal allergy* | 29 | 10.46(7.11-15.38) | 10.44(220.87) | 9.42(6.41) | 3.24(2.32) |
| Graft versus host disease* | 27 | 9.82(6.59-14.62) | 9.81(191.69) | 8.9(5.98) | 3.15(2.22) |
| Acute myeloid leukaemia recurrent* | 26 | 56.02(34.18-91.8) | 55.94(850.35) | 34.3(20.93) | 5.1(3.27) |

Abbreviation: Asterisks (*) indicate relevant signal in algorithm; ROR, reporting odds ratio; PRR, proportional reporting ratio; EBGM, empirical Bayesian geometric mean; EBGM05, the lower limit of the 95% CI of EBGM; IC, information component; IC025, the lower limit of the 95% CI of the IC; CI, confidence interval; PT, preferred term; AEs, adverse events.

**Supplementary Table 12**

Top 50 most frequent adverse events for IVIg at the PT level in patients with body weight 50-100kg from FAERS data.

| PT | Number of reports | ROR (95%Cl) | PRR(X2) | EBGM(EBGM05) | IC(IC025) |
| --- | --- | --- | --- | --- | --- |
| Headache* | 2644 | 2.48(2.39-2.58) | 2.44(2228.76) | 2.41(2.32) | 1.27(1.21) |
| Sinusitis* | 2184 | 13.45(12.87-14.07) | 13.17(22186.08) | 11.97(11.45) | 3.58(3.51) |
| Pyrexia* | 1550 | 2.43(2.31-2.56) | 2.41(1261.09) | 2.38(2.26) | 1.25(1.18) |
| Infusion related reaction* | 1412 | 9.47(8.97-10) | 9.35(9787.71) | 8.75(8.28) | 3.13(3.04) |
| Chills* | 1226 | 5.02(4.74-5.32) | 4.97(3746.99) | 4.82(4.55) | 2.27(2.18) |
| Urticaria* | 1209 | 4.61(4.35-4.89) | 4.57(3256.37) | 4.44(4.19) | 2.15(2.06) |
| Product dose omission issue* | 990 | 2.38(2.23-2.53) | 2.36(766.04) | 2.34(2.19) | 1.22(1.13) |
| Covid-19* | 855 | 4(3.73-4.28) | 3.97(1843.66) | 3.88(3.62) | 1.95(1.85) |
| Urinary tract infection* | 847 | 2.84(2.65-3.04) | 2.82(975.57) | 2.78(2.6) | 1.47(1.37) |
| Bronchitis* | 748 | 5.85(5.43-6.29) | 5.81(2845.46) | 5.59(5.19) | 2.48(2.37) |
| Nasopharyngitis* | 665 | 2.71(2.51-2.93) | 2.7(697.55) | 2.66(2.46) | 1.41(1.3) |
| Infusion site pain* | 648 | 20.75(19.09-22.56) | 20.62(10333.96) | 17.75(16.33) | 4.15(3.99) |
| Inappropriate schedule of product administration* | 604 | 2.28(2.1-2.47) | 2.27(423.67) | 2.25(2.07) | 1.17(1.05) |
| Infusion site erythema* | 602 | 32.97(30.13-36.09) | 32.77(14583.91) | 25.98(23.74) | 4.7(4.51) |
| Upper respiratory tract infection* | 573 | 8.28(7.61-9.01) | 8.24(3412.98) | 7.77(7.14) | 2.96(2.82) |
| Infection* | 570 | 3.22(2.96-3.5) | 3.21(844.57) | 3.15(2.9) | 1.65(1.53) |
| Infusion site swelling* | 565 | 51.04(46.25-56.32) | 50.75(19393.4) | 36.01(32.63) | 5.17(4.94) |
| No adverse event* | 512 | 4.57(4.19-5) | 4.55(1370.16) | 4.42(4.05) | 2.15(2.01) |
| Migraine* | 490 | 3.11(2.85-3.41) | 3.1(681.81) | 3.05(2.79) | 1.61(1.47) |
| Influenza* | 405 | 2.67(2.42-2.94) | 2.66(410.79) | 2.62(2.38) | 1.39(1.24) |
| Illness* | 397 | 4.61(4.17-5.1) | 4.6(1077.74) | 4.47(4.04) | 2.16(2) |
| Ear infection* | 376 | 10.94(9.85-12.16) | 10.91(3103.39) | 10.08(9.07) | 3.33(3.14) |
| Influenza like illness* | 345 | 2.83(2.54-3.15) | 2.82(396.41) | 2.78(2.5) | 1.47(1.31) |
| Infusion site pruritus* | 340 | 69.7(60.99-79.67) | 69.46(14555.86) | 44.43(38.88) | 5.47(5.12) |
| Infusion site extravasation* | 320 | 39.89(35.15-45.27) | 39.77(9094.09) | 30.15(26.57) | 4.91(4.61) |
| Meningitis aseptic* | 309 | 60.66(52.9-69.55) | 60.46(12035.05) | 40.6(35.41) | 5.34(4.98) |
| Respiratory tract infection* | 294 | 8.5(7.55-9.57) | 8.48(1811.96) | 7.98(7.09) | 3(2.79) |
| Anaphylactic reaction* | 281 | 3.53(3.13-3.97) | 3.52(493.39) | 3.45(3.06) | 1.79(1.6) |
| Insurance issue* | 276 | 21.59(18.99-24.55) | 21.53(4585.59) | 18.42(16.2) | 4.2(3.93) |
| Injection site erythema* | 275 | 2.74(2.43-3.09) | 2.74(296.89) | 2.7(2.39) | 1.43(1.25) |
| Oxygen saturation decreased* | 268 | 2.36(2.09-2.66) | 2.35(204.97) | 2.33(2.06) | 1.22(1.03) |
| Seasonal allergy* | 247 | 9.56(8.4-10.88) | 9.54(1749.53) | 8.91(7.83) | 3.16(2.92) |
| Viral infection* | 224 | 4.77(4.18-5.45) | 4.76(641.08) | 4.62(4.04) | 2.21(1.99) |
| Injection site swelling* | 204 | 3.41(2.97-3.92) | 3.41(337.95) | 3.34(2.91) | 1.74(1.52) |
| Infusion site bruising* | 202 | 92.07(76.66-110.58) | 91.88(10304.38) | 52.57(43.77) | 5.72(5.14) |
| Infusion site haemorrhage* | 192 | 24.13(20.66-28.18) | 24.08(3540.81) | 20.24(17.33) | 4.34(3.98) |
| Incorrect drug administration rate* | 185 | 28.39(24.18-33.32) | 28.33(3950.29) | 23.13(19.71) | 4.53(4.14) |
| Body temperature increased* | 183 | 4.13(3.56-4.78) | 4.12(418.41) | 4.02(3.47) | 2.01(1.77) |
| Multiple allergies* | 179 | 15.39(13.17-17.99) | 15.37(2132.87) | 13.74(11.76) | 3.78(3.45) |
| Cystitis* | 175 | 3.07(2.65-3.57) | 3.07(238.33) | 3.02(2.6) | 1.59(1.36) |
| Fungal infection* | 161 | 3.3(2.82-3.86) | 3.3(251.01) | 3.24(2.77) | 1.69(1.44) |
| Sinus disorder* | 152 | 4.62(3.93-5.43) | 4.61(414.54) | 4.48(3.81) | 2.16(1.89) |
| Staphylococcal infection* | 150 | 2.8(2.38-3.29) | 2.79(168.93) | 2.75(2.34) | 1.46(1.21) |
| Gastroenteritis viral* | 137 | 5.71(4.81-6.77) | 5.7(507.13) | 5.49(4.62) | 2.46(2.16) |
| Infusion site mass* | 132 | 137.35(107.02-176.28) | 137.16(8345.91) | 64.69(50.4) | 6.02(5.14) |
| Kidney infection* | 127 | 4.48(3.75-5.35) | 4.47(330.37) | 4.35(3.64) | 2.12(1.82) |
| Dermatitis contact* | 127 | 5.39(4.51-6.44) | 5.38(434.15) | 5.2(4.35) | 2.38(2.07) |
| Poor venous access* | 126 | 11.11(9.25-13.33) | 11.09(1059.81) | 10.24(8.53) | 3.36(2.99) |
| Localised infection* | 125 | 3.72(3.12-4.45) | 3.72(241.28) | 3.64(3.05) | 1.86(1.57) |
| Skin infection* | 122 | 7(5.83-8.4) | 6.99(592.31) | 6.66(5.55) | 2.74(2.4) |

Abbreviation: Asterisks (*) indicate relevant signal in algorithm; ROR, reporting odds ratio; PRR, proportional reporting ratio; EBGM, empirical Bayesian geometric mean; EBGM05, the lower limit of the 95% CI of EBGM; IC, information component; IC025, the lower limit of the 95% CI of the IC; CI, confidence interval; PT, preferred term; AEs, adverse events.

**Supplementary Table 13**

Top 50 most frequent adverse events for IVIg at the PT level in patients with body weight >100kg from FAERS data.

| PT | Number of reports | ROR (95%Cl) | PRR(X2) | EBGM(EBGM05) | IC(IC025) |
| --- | --- | --- | --- | --- | --- |
| Headache* | 544 | 2.83(2.6-3.08) | 2.78(609.17) | 2.73(2.51) | 1.45(1.32) |
| Sinusitis* | 453 | 12.81(11.61-14.13) | 12.52(4322.21) | 11.35(10.29) | 3.5(3.33) |
| Pruritus* | 295 | 2.51(2.23-2.82) | 2.49(257.75) | 2.45(2.18) | 1.29(1.12) |
| Infusion related reaction* | 288 | 9.87(8.74-11.14) | 9.73(2076.2) | 9.02(7.99) | 3.17(2.96) |
| Urticaria* | 269 | 5.16(4.57-5.84) | 5.1(850.82) | 4.92(4.35) | 2.3(2.1) |
| Pyrexia* | 264 | 2.86(2.53-3.23) | 2.83(306.01) | 2.78(2.46) | 1.48(1.29) |
| Chills* | 240 | 5.35(4.7-6.09) | 5.29(799.6) | 5.1(4.47) | 2.35(2.13) |
| Bronchitis* | 178 | 5.72(4.92-6.66) | 5.68(653.78) | 5.45(4.68) | 2.45(2.19) |
| Urinary tract infection* | 160 | 3.03(2.58-3.54) | 3.01(209.33) | 2.95(2.52) | 1.56(1.31) |
| Upper respiratory tract infection* | 122 | 7.07(5.88-8.49) | 7.03(593.63) | 6.67(5.55) | 2.74(2.4) |
| Migraine* | 104 | 3.85(3.17-4.69) | 3.84(211.18) | 3.74(3.08) | 1.9(1.58) |
| No adverse event* | 104 | 3.75(3.08-4.56) | 3.73(201.57) | 3.64(3) | 1.87(1.54) |
| Infusion site pain* | 100 | 15.92(12.9-19.64) | 15.84(1216.31) | 13.98(11.33) | 3.81(3.32) |
| Infusion site erythema* | 92 | 22.61(18.06-28.3) | 22.5(1570.85) | 18.86(15.07) | 4.24(3.66) |
| Ear infection* | 91 | 10.72(8.64-13.29) | 10.67(727.47) | 9.82(7.91) | 3.3(2.85) |
| Infection* | 90 | 2.61(2.12-3.22) | 2.6(86.82) | 2.56(2.08) | 1.36(1.03) |
| Influenza* | 79 | 2.58(2.06-3.22) | 2.57(74.17) | 2.53(2.03) | 1.34(0.99) |
| Illness* | 77 | 3.72(2.96-4.67) | 3.71(147.35) | 3.62(2.88) | 1.86(1.47) |
| Infusion site extravasation* | 77 | 38.5(29.7-49.91) | 38.34(2079.46) | 28.73(22.16) | 4.84(4.03) |
| Infusion site swelling* | 70 | 29.99(23.02-39.06) | 29.88(1538.26) | 23.73(18.22) | 4.57(3.79) |
| Infusion site pruritus* | 63 | 62.39(45.81-84.98) | 62.18(2427.33) | 40.16(29.48) | 5.33(4.21) |
| Insurance issue* | 56 | 16.08(12.15-21.29) | 16.04(689.69) | 14.13(10.67) | 3.82(3.11) |
| Respiratory tract infection* | 56 | 8.39(6.39-11.01) | 8.37(337.74) | 7.85(5.98) | 2.97(2.41) |
| Meningitis aseptic* | 55 | 89.67(62.83-127.99) | 89.41(2658.33) | 49.88(34.95) | 5.64(4.26) |
| Viral infection* | 45 | 5.29(3.92-7.14) | 5.28(149.11) | 5.09(3.77) | 2.35(1.79) |
| Incorrect drug administration rate* | 45 | 36.94(26.35-51.77) | 36.85(1177.13) | 27.89(19.89) | 4.8(3.66) |
| Cystitis* | 44 | 4.54(3.36-6.14) | 4.53(116.28) | 4.39(3.25) | 2.13(1.59) |
| Infusion site haemorrhage* | 42 | 28.03(19.97-39.34) | 27.97(871.74) | 22.52(16.05) | 4.49(3.42) |
| Seasonal allergy* | 40 | 6.88(5-9.47) | 6.87(188.81) | 6.52(4.74) | 2.71(2.06) |
| Multiple allergies* | 39 | 17.35(12.37-24.32) | 17.31(518.39) | 15.1(10.77) | 3.92(2.99) |
| Localised infection* | 39 | 3.5(2.54-4.81) | 3.49(67.23) | 3.41(2.48) | 1.77(1.22) |
| Fungal infection* | 37 | 3.27(2.36-4.54) | 3.27(56.65) | 3.2(2.31) | 1.68(1.12) |
| Gastroenteritis viral* | 36 | 5.71(4.08-7.99) | 5.7(132.77) | 5.47(3.91) | 2.45(1.8) |
| Kidney infection* | 33 | 4.78(3.37-6.78) | 4.77(94.44) | 4.62(3.26) | 2.21(1.55) |
| Lower respiratory tract infection* | 33 | 3.41(2.41-4.82) | 3.4(54.35) | 3.33(2.36) | 1.74(1.14) |
| Body temperature increased* | 30 | 3.84(2.67-5.53) | 3.84(60.88) | 3.74(2.6) | 1.9(1.25) |
| Infusion site bruising* | 29 | 68.31(42.99-108.54) | 68.21(1187.68) | 42.56(26.79) | 5.41(3.53) |
| Dermatitis contact* | 27 | 5.66(3.84-8.33) | 5.65(98.4) | 5.43(3.69) | 2.44(1.67) |
| Photophobia* | 25 | 4.48(3.01-6.69) | 4.48(64.95) | 4.34(2.91) | 2.12(1.36) |
| Product distribution issue* | 25 | 27.67(17.85-42.9) | 27.64(513.47) | 22.31(14.39) | 4.48(2.99) |
| Sinus disorder* | 24 | 3.35(2.23-5.03) | 3.35(38.41) | 3.28(2.19) | 1.71(1) |
| Haemolysis* | 24 | 18.58(12.05-28.63) | 18.55(341.31) | 16.03(10.4) | 4(2.7) |
| Skin infection* | 24 | 5.03(3.34-7.57) | 5.02(74.02) | 4.85(3.22) | 2.28(1.48) |
| Infusion site discharge* | 24 | 24.83(15.94-38.66) | 24.79(447.67) | 20.44(13.12) | 4.35(2.89) |
| Infusion site reaction* | 23 | 28.6(18.08-45.25) | 28.57(486.23) | 22.91(14.48) | 4.52(2.93) |
| Poor venous access* | 23 | 8.08(5.29-12.34) | 8.07(132.82) | 7.59(4.97) | 2.92(1.96) |
| Infusion site rash* | 23 | 48.95(29.96-79.99) | 48.89(748.16) | 34.21(20.93) | 5.1(3.16) |
| Tooth infection* | 22 | 4.71(3.07-7.22) | 4.7(61.56) | 4.55(2.97) | 2.19(1.36) |
| Weight fluctuation* | 22 | 3.8(2.48-5.81) | 3.79(43.78) | 3.7(2.42) | 1.89(1.11) |
| Tachypnoea* | 21 | 5.1(3.29-7.89) | 5.09(66.01) | 4.91(3.17) | 2.3(1.43) |

Abbreviation: Asterisks (*) indicate relevant signal in algorithm; ROR, reporting odds ratio; PRR, proportional reporting ratio; EBGM, empirical Bayesian geometric mean; EBGM05, the lower limit of the 95% CI of EBGM; IC, information component; IC025, the lower limit of the 95% CI of the IC; CI, confidence interval; PT, preferred term; AEs, adverse events.

**Supplementary Table 14**

Top 50 most frequent adverse events for IVIg at the PT level in patients reported by medical personnels from FAERS data.

| PT | Number of reports | ROR (95%Cl) | PRR(X2) | EBGM(EBGM05) | IC(IC025) |
| --- | --- | --- | --- | --- | --- |
| Headache* | 3437 | 3.19(3.08-3.3) | 3.14(4938.69) | 3.09(2.99) | 1.63(1.58) |
| Pyrexia* | 2539 | 2.72(2.61-2.83) | 2.68(2654.22) | 2.65(2.55) | 1.41(1.35) |
| No adverse event* | 2098 | 4.87(4.66-5.08) | 4.81(6147.26) | 4.69(4.49) | 2.23(2.16) |
| Pruritus* | 1927 | 2.38(2.27-2.49) | 2.36(1493.17) | 2.34(2.23) | 1.22(1.16) |
| Urticaria* | 1803 | 4.95(4.72-5.19) | 4.9(5431.64) | 4.77(4.55) | 2.26(2.18) |
| Infusion related reaction* | 1649 | 7.35(7-7.73) | 7.28(8525.81) | 6.98(6.64) | 2.8(2.73) |
| Chills* | 1611 | 6.43(6.12-6.76) | 6.37(7000.33) | 6.15(5.84) | 2.62(2.54) |
| Sinusitis* | 1607 | 7.87(7.48-8.28) | 7.79(9050.11) | 7.45(7.08) | 2.9(2.82) |
| Infection* | 1046 | 2.9(2.73-3.08) | 2.89(1267.37) | 2.85(2.68) | 1.51(1.42) |
| Infusion site pain* | 732 | 44.17(40.66-47.98) | 43.94(23669.07) | 34.08(31.38) | 5.09(4.91) |
| Infusion site erythema* | 713 | 74.27(67.88-81.28) | 73.9(34161.07) | 49.56(45.29) | 5.63(5.41) |
| Infusion site swelling* | 681 | 90.36(82.13-99.4) | 89.92(37202.42) | 56.24(51.12) | 5.81(5.57) |
| Blood pressure increased* | 680 | 2.29(2.13-2.48) | 2.29(486.8) | 2.27(2.1) | 1.18(1.07) |
| Bronchitis* | 593 | 3.9(3.59-4.23) | 3.89(1239.84) | 3.81(3.51) | 1.93(1.8) |
| Illness* | 564 | 4.12(3.79-4.48) | 4.11(1291.52) | 4.02(3.7) | 2.01(1.88) |
| Chest discomfort* | 521 | 2.35(2.16-2.57) | 2.35(397.47) | 2.33(2.13) | 1.22(1.09) |
| Meningitis aseptic* | 501 | 40.79(36.94-45.03) | 40.64(15188.96) | 32.08(29.05) | 5(4.77) |
| Migraine* | 497 | 3.48(3.18-3.8) | 3.47(854.82) | 3.41(3.12) | 1.77(1.63) |
| Anaphylactic reaction* | 492 | 2.8(2.56-3.07) | 2.8(558.66) | 2.76(2.53) | 1.47(1.33) |
| Upper respiratory tract infection* | 441 | 3.97(3.61-4.37) | 3.96(952.26) | 3.89(3.53) | 1.96(1.81) |
| Infusion site pruritus* | 433 | 144.93(126.93-165.49) | 144.48(31167.09) | 73.48(64.35) | 6.2(5.81) |
| Infusion site extravasation* | 418 | 24.01(21.65-26.63) | 23.94(7906.64) | 20.74(18.7) | 4.37(4.16) |
| Oxygen saturation decreased* | 404 | 2.66(2.41-2.93) | 2.65(408.71) | 2.62(2.38) | 1.39(1.24) |
| Insurance issue* | 403 | 20.39(18.37-22.63) | 20.33(6511.76) | 17.99(16.21) | 4.17(3.96) |
| Drug ineffective for unapproved indication* | 387 | 2.94(2.66-3.26) | 2.94(485.47) | 2.9(2.62) | 1.54(1.38) |
| Ear infection* | 372 | 7.67(6.91-8.51) | 7.65(2045.75) | 7.32(6.6) | 2.87(2.69) |
| Haemolytic anaemia* | 352 | 11.32(10.16-12.62) | 11.3(3068.93) | 10.56(9.48) | 3.4(3.2) |
| Influenza like illness* | 346 | 2.52(2.26-2.8) | 2.51(310.64) | 2.49(2.24) | 1.32(1.15) |
| Therapy interrupted* | 323 | 2.59(2.32-2.89) | 2.59(309.88) | 2.56(2.29) | 1.36(1.19) |
| Haemolysis* | 257 | 11.89(10.47-13.5) | 11.87(2367.42) | 11.06(9.74) | 3.47(3.23) |
| Body temperature increased* | 249 | 4.87(4.29-5.53) | 4.86(739.92) | 4.74(4.18) | 2.24(2.04) |
| Respiratory tract infection* | 248 | 3.63(3.2-4.12) | 3.63(461.36) | 3.57(3.14) | 1.83(1.63) |
| Recalled product administered* | 229 | 46.09(39.73-53.46) | 46.01(7686.4) | 35.31(30.44) | 5.14(4.73) |
| Infusion site haemorrhage* | 228 | 68.46(58.5-80.1) | 68.35(10339.33) | 47.02(40.18) | 5.56(5.07) |
| Infusion site bruising* | 227 | 110.67(93.17-131.46) | 110.49(14080.98) | 63.6(53.54) | 5.99(5.41) |
| Viral infection* | 223 | 3.34(2.92-3.81) | 3.34(356.8) | 3.28(2.88) | 1.72(1.51) |
| Incorrect drug administration rate* | 221 | 24.53(21.27-28.28) | 24.49(4270.26) | 21.14(18.34) | 4.4(4.07) |
| Cytomegalovirus infection* | 208 | 3.42(2.98-3.93) | 3.42(347.96) | 3.36(2.93) | 1.75(1.53) |
| Tachypnoea* | 197 | 4.23(3.67-4.87) | 4.22(470.82) | 4.13(3.59) | 2.05(1.82) |
| Staphylococcal infection* | 193 | 2.38(2.06-2.74) | 2.37(151.08) | 2.35(2.04) | 1.23(1.01) |
| Serum sickness* | 192 | 20.8(17.88-24.2) | 20.78(3168.1) | 18.33(15.76) | 4.2(3.85) |
| Poor venous access* | 176 | 7.38(6.34-8.58) | 7.37(923.04) | 7.07(6.07) | 2.82(2.55) |
| Feeling cold* | 163 | 3.74(3.2-4.37) | 3.74(319.02) | 3.67(3.14) | 1.88(1.62) |
| Seasonal allergy* | 162 | 6.74(5.76-7.89) | 6.73(756.29) | 6.48(5.54) | 2.7(2.42) |
| Chronic inflammatory demyelinating polyradiculoneuropathy* | 159 | 51.37(42.88-61.53) | 51.31(5819.35) | 38.33(32) | 5.26(4.7) |
| Syringe issue* | 156 | 2.38(2.03-2.78) | 2.37(122.25) | 2.35(2.01) | 1.23(0.99) |
| Acute graft versus host disease in skin* | 153 | 16.22(13.72-19.16) | 16.2(1966.08) | 14.69(12.43) | 3.88(3.51) |
| Infusion site reaction* | 150 | 28.91(24.27-34.44) | 28.88(3376.3) | 24.31(20.41) | 4.6(4.14) |
| Gastroenteritis viral* | 146 | 4.7(3.99-5.55) | 4.7(412.28) | 4.59(3.89) | 2.2(1.92) |
| Multiple allergies* | 138 | 12.04(10.12-14.32) | 12.03(1290.33) | 11.2(9.41) | 3.49(3.13) |

Abbreviation: Asterisks (*) indicate relevant signal in algorithm; ROR, reporting odds ratio; PRR, proportional reporting ratio; EBGM, empirical Bayesian geometric mean; EBGM05, the lower limit of the 95% CI of EBGM; IC, information component; IC025, the lower limit of the 95% CI of the IC; CI, confidence interval; PT, preferred term; AEs, adverse events.

**Supplementary Table 15**

Top 50 most frequent adverse events for IVIg at the PT level in patients reported by non-medical personnels from FAERS data.

| PT | Number of reports | ROR (95%Cl) | PRR(X2) | EBGM(EBGM05) | IC(IC025) |
| --- | --- | --- | --- | --- | --- |
| Headache* | 2699 | 2.43(2.34-2.52) | 2.39(2180.95) | 2.37(2.28) | 1.25(1.19) |
| Sinusitis* | 1689 | 10.74(10.23-11.28) | 10.56(14089.1) | 10.2(9.71) | 3.35(3.27) |
| Pneumonia* | 1286 | 3.04(2.88-3.22) | 3.02(1721.5) | 2.99(2.83) | 1.58(1.5) |
| Pyrexia* | 1252 | 3.2(3.03-3.39) | 3.17(1846.55) | 3.14(2.97) | 1.65(1.57) |
| No adverse event* | 948 | 3.44(3.23-3.67) | 3.42(1605.01) | 3.39(3.18) | 1.76(1.66) |
| Infection* | 759 | 4.37(4.07-4.7) | 4.34(1925.55) | 4.29(3.99) | 2.1(1.99) |
| Infusion site pain* | 753 | 45.49(42.1-49.15) | 45.12(27829.42) | 38.79(35.9) | 5.28(5.09) |
| Chills* | 671 | 3.9(3.62-4.21) | 3.88(1417.36) | 3.84(3.56) | 1.94(1.82) |
| Infusion site swelling* | 647 | 122.98(112.05-134.99) | 122.11(53478.28) | 84.33(76.83) | 6.4(6.09) |
| Bronchitis* | 631 | 5.24(4.84-5.67) | 5.21(2106.43) | 5.13(4.74) | 2.36(2.23) |
| Infusion site erythema* | 627 | 75.5(69.09-82.51) | 74.99(35806.87) | 58.87(53.87) | 5.88(5.62) |
| Migraine* | 626 | 3.44(3.18-3.73) | 3.43(1064.48) | 3.4(3.14) | 1.76(1.64) |
| Urinary tract infection* | 614 | 2.44(2.25-2.64) | 2.43(513.17) | 2.42(2.23) | 1.27(1.15) |
| Infusion related reaction* | 594 | 17.71(16.29-19.24) | 17.6(8730.96) | 16.58(15.25) | 4.05(3.89) |
| Urticaria* | 534 | 2.33(2.14-2.53) | 2.32(397.78) | 2.31(2.12) | 1.21(1.08) |
| Illness* | 472 | 2.73(2.5-3) | 2.73(511.58) | 2.71(2.47) | 1.44(1.3) |
| Influenza* | 432 | 2.23(2.03-2.46) | 2.23(290.48) | 2.22(2.02) | 1.15(1.01) |
| Upper respiratory tract infection* | 389 | 6.7(6.06-7.42) | 6.68(1834.48) | 6.54(5.92) | 2.71(2.54) |
| Influenza like illness* | 368 | 2.32(2.1-2.57) | 2.32(273.54) | 2.31(2.08) | 1.21(1.05) |
| Haemorrhage* | 362 | 2.3(2.07-2.55) | 2.29(262.63) | 2.28(2.06) | 1.19(1.03) |
| Infusion site extravasation* | 362 | 98.38(87.21-110.97) | 97.99(25482.06) | 72.11(63.93) | 6.17(5.74) |
| Infusion site pruritus* | 334 | 141.51(123.95-161.57) | 141(30475.07) | 92.89(81.36) | 6.54(6) |
| Ear infection* | 320 | 7.74(6.93-8.65) | 7.72(1820.32) | 7.53(6.74) | 2.91(2.72) |
| Meningitis aseptic* | 319 | 311.26(264.95-365.67) | 310.17(45690.28) | 144.69(123.16) | 7.18(6.44) |
| Insurance issue* | 310 | 17.15(15.29-19.24) | 17.1(4419.11) | 16.14(14.38) | 4.01(3.77) |
| Therapy interrupted* | 247 | 3.81(3.36-4.32) | 3.8(502.84) | 3.76(3.32) | 1.91(1.71) |
| Respiratory tract infection* | 243 | 8.59(7.56-9.76) | 8.57(1574.69) | 8.33(7.33) | 3.06(2.83) |
| Viral infection* | 237 | 4.78(4.2-5.43) | 4.77(694.02) | 4.7(4.14) | 2.23(2.02) |
| Incorrect drug administration rate* | 227 | 59.97(51.93-69.26) | 59.82(10743.66) | 49.13(42.54) | 5.62(5.13) |
| Infusion site bruising* | 225 | 104.56(89.63-121.97) | 104.3(16594.32) | 75.46(64.69) | 6.24(5.61) |
| Infusion site haemorrhage* | 221 | 51.04(44.2-58.94) | 50.92(9095.96) | 42.98(37.22) | 5.43(4.97) |
| Anaphylactic reaction* | 205 | 5.34(4.65-6.13) | 5.33(707.5) | 5.25(4.57) | 2.39(2.16) |
| Herpes zoster* | 193 | 2.33(2.02-2.69) | 2.33(145.05) | 2.32(2.01) | 1.21(0.99) |
| Cellulitis* | 174 | 2.87(2.47-3.33) | 2.86(208.85) | 2.84(2.45) | 1.51(1.27) |
| Blood immunoglobulin g decreased* | 168 | 96.04(80.52-114.54) | 95.86(11631.21) | 70.96(59.5) | 6.15(5.4) |
| Lower respiratory tract infection* | 165 | 2.69(2.31-3.13) | 2.69(172.92) | 2.67(2.29) | 1.42(1.18) |
| Poor venous access* | 161 | 11.47(9.8-13.43) | 11.45(1473.61) | 11.03(9.42) | 3.46(3.14) |
| Infusion site mass* | 160 | 215.84(175.31-265.75) | 215.46(18975.2) | 120.15(97.58) | 6.91(5.83) |
| Staphylococcal infection* | 149 | 3.63(3.08-4.26) | 3.62(279.08) | 3.59(3.05) | 1.84(1.58) |
| Haemarthrosis* | 144 | 13.11(11.09-15.5) | 13.09(1534.1) | 12.53(10.6) | 3.65(3.29) |
| Sinus disorder* | 143 | 3.89(3.3-4.59) | 3.88(301.95) | 3.84(3.26) | 1.94(1.67) |
| Cystitis* | 136 | 2.46(2.08-2.91) | 2.46(116.42) | 2.44(2.06) | 1.29(1.03) |
| Gastroenteritis viral* | 132 | 4.29(3.61-5.1) | 4.29(327.5) | 4.23(3.57) | 2.08(1.8) |
| Product availability issue* | 127 | 2.74(2.3-3.26) | 2.74(138.79) | 2.72(2.28) | 1.44(1.17) |
| Infusion site reaction* | 123 | 51.19(42.21-62.08) | 51.12(5080.35) | 43.13(35.56) | 5.43(4.73) |
| Needle issue* | 121 | 2.75(2.3-3.29) | 2.75(133.27) | 2.73(2.28) | 1.45(1.17) |
| Multiple allergies* | 121 | 9.33(7.78-11.18) | 9.32(868.37) | 9.04(7.54) | 3.18(2.82) |
| Kidney infection* | 119 | 3.31(2.76-3.97) | 3.31(189.26) | 3.28(2.74) | 1.71(1.42) |
| Recalled product administered* | 118 | 7.4(6.16-8.88) | 7.39(634.6) | 7.22(6.01) | 2.85(2.51) |
| Localised infection* | 114 | 2.7(2.24-3.24) | 2.69(120.33) | 2.68(2.23) | 1.42(1.13) |

Abbreviation: Asterisks (*) indicate relevant signal in algorithm; ROR, reporting odds ratio; PRR, proportional reporting ratio; EBGM, empirical Bayesian geometric mean; EBGM05, the lower limit of the 95% CI of EBGM; IC, information component; IC025, the lower limit of the 95% CI of the IC; CI, confidence interval; PT, preferred term; AEs, adverse events.

**Supplementary Table 16**

Top 50 most frequent adverse events for IVIg excluding common medication co-usage at the PT level from FAERS data.

| PT | Number of reports | ROR (95%Cl) | PRR(X2) | EBGM(EBGM05) | IC(IC025) |
| --- | --- | --- | --- | --- | --- |
| Pyrexia* | 1667 | 4.09(3.9-4.3) | 4.02(3789.73) | 4.01(3.82) | 2(1.93) |
| Chills* | 727 | 5.26(4.89-5.66) | 5.22(2466.85) | 5.19(4.82) | 2.38(2.26) |
| Sinusitis* | 678 | 5.81(5.38-6.27) | 5.77(2654.24) | 5.73(5.31) | 2.52(2.4) |
| Infusion related reaction* | 528 | 7.37(6.76-8.03) | 7.33(2858.32) | 7.26(6.66) | 2.86(2.72) |
| Haemorrhage* | 502 | 4.08(3.74-4.46) | 4.06(1154.64) | 4.05(3.71) | 2.02(1.88) |
| Covid-19* | 493 | 2.35(2.15-2.57) | 2.34(379.29) | 2.34(2.14) | 1.23(1.09) |
| Infection* | 485 | 2.92(2.67-3.19) | 2.91(605.64) | 2.9(2.65) | 1.54(1.4) |
| Febrile neutropenia* | 403 | 5.18(4.69-5.71) | 5.16(1341.99) | 5.13(4.65) | 2.36(2.2) |
| Infusion site pain* | 359 | 31.22(28.08-34.7) | 31.07(10029.42) | 29.86(26.86) | 4.9(4.63) |
| Infusion site erythema* | 347 | 56.05(50.24-62.53) | 55.79(17366.46) | 51.96(46.57) | 5.7(5.34) |
| Sepsis* | 320 | 2.38(2.13-2.65) | 2.37(252.97) | 2.37(2.12) | 1.24(1.07) |
| Tachycardia* | 316 | 2.98(2.67-3.33) | 2.97(412.67) | 2.97(2.65) | 1.57(1.4) |
| Infusion site swelling* | 302 | 73.14(64.97-82.33) | 72.84(19485.06) | 66.41(59) | 6.05(5.6) |
| Serum sickness* | 284 | 94.29(83.32-106.7) | 93.93(23176.11) | 83.48(73.77) | 6.38(5.84) |
| Bronchitis* | 271 | 3.02(2.68-3.41) | 3.01(363.8) | 3.01(2.67) | 1.59(1.4) |
| Cytomegalovirus infection* | 270 | 13.79(12.22-15.56) | 13.74(3132.8) | 13.51(11.98) | 3.76(3.51) |
| Meningitis aseptic* | 249 | 65.92(57.9-75.06) | 65.7(14574.9) | 60.44(53.08) | 5.92(5.42) |
| Multiple organ dysfunction syndrome* | 242 | 4.47(3.94-5.07) | 4.46(645.95) | 4.44(3.91) | 2.15(1.94) |
| Respiratory failure* | 229 | 2.57(2.26-2.93) | 2.57(218.61) | 2.56(2.25) | 1.36(1.16) |
| Haemarthrosis* | 220 | 23.25(20.32-26.59) | 23.18(4527.95) | 22.51(19.68) | 4.49(4.16) |
| Infusion site extravasation* | 213 | 34.99(30.49-40.15) | 34.89(6697.07) | 33.37(29.08) | 5.06(4.65) |
| Oxygen saturation decreased* | 212 | 3.31(2.9-3.79) | 3.31(340.2) | 3.3(2.88) | 1.72(1.51) |
| Anaphylactic reaction* | 210 | 3.35(2.93-3.84) | 3.34(343.96) | 3.33(2.91) | 1.74(1.52) |
| Haemolytic anaemia* | 202 | 19.74(17.16-22.7) | 19.69(3490.45) | 19.2(16.69) | 4.26(3.93) |
| Transplant rejection* | 201 | 21.52(18.7-24.76) | 21.46(3811.8) | 20.89(18.15) | 4.38(4.04) |
| Haemolysis* | 189 | 21.69(18.76-25.07) | 21.64(3614.75) | 21.05(18.21) | 4.4(4.04) |
| Upper respiratory tract infection* | 177 | 3.31(2.86-3.84) | 3.31(283.85) | 3.3(2.84) | 1.72(1.49) |
| Acute graft versus host disease in skin* | 175 | 59.15(50.7-69.01) | 59.01(9244.73) | 54.74(46.92) | 5.77(5.16) |
| Acute graft versus host disease* | 167 | 30.86(26.43-36.03) | 30.79(4621.98) | 29.6(25.35) | 4.89(4.43) |
| Syringe issue* | 161 | 7.96(6.81-9.3) | 7.94(967.16) | 7.87(6.74) | 2.98(2.69) |
| Pulmonary oedema* | 153 | 2.8(2.39-3.28) | 2.8(176.07) | 2.79(2.38) | 1.48(1.23) |
| Epstein-barr virus infection* | 153 | 21.62(18.41-25.39) | 21.58(2917.64) | 20.99(17.87) | 4.39(3.98) |
| Infusion site pruritus* | 150 | 83.95(70.9-99.41) | 83.78(11023.67) | 75.38(63.66) | 6.24(5.41) |
| Septic shock* | 148 | 2.9(2.47-3.41) | 2.9(183.4) | 2.89(2.46) | 1.53(1.28) |
| Hypoxia* | 147 | 3.54(3.01-4.17) | 3.54(266.6) | 3.53(3) | 1.82(1.56) |
| Cytomegalovirus viraemia* | 140 | 29.88(25.23-35.39) | 29.83(3749.89) | 28.71(24.25) | 4.84(4.34) |
| Post transplant lymphoproliferative disorder* | 140 | 25.73(21.74-30.46) | 25.69(3210.42) | 24.86(21) | 4.64(4.16) |
| Insurance issue* | 139 | 11.89(10.06-14.06) | 11.87(1362.37) | 11.7(9.89) | 3.55(3.2) |
| Kidney transplant rejection* | 138 | 23.63(19.94-28) | 23.59(2893.45) | 22.89(19.32) | 4.52(4.06) |
| Cytomegalovirus test positive* | 135 | 56.13(47.11-66.87) | 56.03(6783.5) | 52.16(43.78) | 5.7(4.99) |
| Thrombotic microangiopathy* | 134 | 12.22(10.3-14.5) | 12.2(1355.9) | 12.02(10.13) | 3.59(3.22) |
| Staphylococcal infection* | 130 | 3.21(2.71-3.82) | 3.21(197.13) | 3.2(2.69) | 1.68(1.4) |
| Graft versus host disease* | 127 | 14.49(12.15-17.27) | 14.46(1561.47) | 14.21(11.92) | 3.83(3.43) |
| Ear infection* | 119 | 4.02(3.36-4.82) | 4.02(268.58) | 4(3.34) | 2(1.7) |
| Viral infection* | 117 | 3.08(2.57-3.7) | 3.08(163.85) | 3.07(2.56) | 1.62(1.33) |
| Blood bilirubin increased* | 115 | 3.44(2.86-4.13) | 3.44(197.88) | 3.43(2.85) | 1.78(1.48) |
| Body temperature increased* | 112 | 4.53(3.76-5.45) | 4.52(305.5) | 4.5(3.74) | 2.17(1.85) |
| Disseminated intravascular coagulation* | 110 | 6.28(5.21-7.58) | 6.27(483.72) | 6.23(5.16) | 2.64(2.3) |
| Cystitis haemorrhagic* | 108 | 23.18(19.14-28.08) | 23.15(2219.63) | 22.48(18.56) | 4.49(3.95) |
| Respiratory tract infection* | 108 | 3.73(3.09-4.51) | 3.72(214.29) | 3.71(3.07) | 1.89(1.58) |

Abbreviation: Asterisks (*) indicate relevant signal in algorithm; ROR, reporting odds ratio; PRR, proportional reporting ratio; EBGM, empirical Bayesian geometric mean; EBGM05, the lower limit of the 95% CI of EBGM; IC, information component; IC025, the lower limit of the 95% CI of the IC; CI, confidence interval; PT, preferred term; AEs, adverse events.
